# Extended Stability Window in Water-in-Salt Electrolytes: Understanding the Origins

**DOI:** 10.1021/jacs.5c12989

**Published:** 2025-09-18

**Authors:** Dario Gomez Vazquez, Johannes Ingenmey, Katharina Trapp, Dennis Ciliak, Mathieu Salanne, Maria R. Lukatskaya

**Affiliations:** † Department of Mechanical and Process Engineering, 111842 ETH Zurich, 8092 Zurich, Switzerland; ‡ Physico-Chimie des Électrolytes et Nanosystèmes Interfaciaux, PHENIX, 27063 Sorbonne Université, CNRS, F-75005 Paris, France; § Institut Universitaire de France (IUF), 75231 Paris Cedex 05, France; ∥ Mulliken Center for Theoretical Chemistry, University of Bonn, 53115 Bonn, Germany

## Abstract

Water-in-salt electrolytes extend the voltage stability range beyond that of dilute systems, enabling the use of high-voltage materials in aqueous energy storage. This stability is often attributed to the formation of a solid electrolyte interphase (SEI) or reduced water activity. However, by studying the hydrogen evolution reaction (HER) on platinum in NaClO_4_ electrolytes (1–17 molal) using electrochemical measurements, MD simulations, and DFT calculations, we show that alternative mechanisms strongly influence the stability window. Specifically, we disentangle the effects from water activity, the local pH increase, and kinetic and transport limitations arising from disrupted hydrogen bonding and sluggish water transport. We observe a nearly linear relationship between the decrease in the surface water coverage and HER exchange current density. As a result, the HER kinetics is slower: with a 7 times decrease in the exchange current density and a 1.5 times increase in the Tafel slope in 17 m solution compared to 1 m. Our MD simulations further reveal that sluggish water transport within the double layer significantly limits the HER, extending the experimental stability window. Ultimately, in this non-SEI-forming electrolyte, reduced bulk water activity plays only a relatively minor role in enhancing the stability of water-in-salt systems.

## Graphical abstract

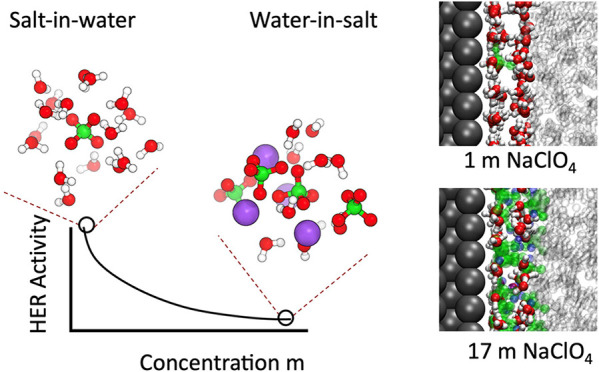

## Introduction

As the use of batteries in applications ranging from portables and electric vehicles to grid storage grows exponentially, safety, cost, and relevant environmental footprint are becoming increasingly important considerations.^1–3^ While batteries with organic electrolytes can achieve excellent energy densities, their intrinsic flammability and toxicity pose significant safety risks.^4,5^ Water-based batteries can offer a safer and greener alternative, but their application is constrained by energy density limitations^4–6^ due to the inherently narrow theoretical stability voltage window of water, which stands at 1.23 V.^6–8^

In recent years, so-called water-in-salt electrolytes (WISE) have sparked a lot of interest in energy community thanks to the extended stability voltage window >3 V that these aqueous systems can provide, thus enabling energy density approaching those of nonaqueous batteries.^6,8,9^ WISE are solutions in which the amount of dissolved salt by weight or volume is larger than the amount of water that these salts are dissolved in.^7,9^ Compared to low or intermediate concentration, in WISE, the environment of the water molecules shifts from water-rich to ion-rich, resulting in altered water activity^7^ and disruption of its hydrogen bonding network (H-bonding).^7,10^ These changes in the bulk solution structure also affect speciation at the electrode–electrolyte interface and overpotentials for water splitting.^7,11^ As such, in WISE, a delayed onset of the hydrogen evolution reaction (HER) at battery anodes by as much as −1.7 V (vs SHE) is observed, enabling utilization of anode materials like carbon-coated TiO_2_ and Li_4_Ti_5_O_12_ in water-based electrolytes.^7^

Many reports attribute the delayed onset of hydrogen evolution to the formation of a passivating solid electrolyte interface (SEI) through the reduction of salt anions in WISE.^6,11–14^ This can take place when WISE is based on the salts of organic imides, such as LiFSI, LiTFSI, and LiBETI.^15–18^ For instance, in a 21m LiTFSI, LiF derived from anion reduction was shown to passivate the electrode surface during the first cycle, leading to suppressed HER in subsequent cycles.^16,19^ Cheng and co-workers attributed this reactivity to a change in the redox potential of the TFSI anion under WISE conditions.^16^ As estimated from machine learning-based molecular dynamics (MD) simulations, the altered local environment changes the relative redox levels of TFSI^–^ anions and H_2_O, favoring anion reduction with respect to the case of dilute solutions.^16,19^ However, experimental investigations have shown that the formation of H_2_ at the electrode under reductive conditions might cause local pH change and thus can play a decisive role in the formation of the SEI.^19^ Ab initio MD simulations have indeed characterized the degradation pathway of TFSI in the presence of hydroxide anions that can lead to the formation of LiF.^20^ More recently, it was demonstrated that the presence of Ag impurities in commonly used LiTFSI electrolytes (98% purity) can modify the electrode surface and thus severely hamper HER kinetics in WISE.^21^ Overall, in such a WISE, decoupling the influence of the vastly different electrolyte structure at high concentration on water reduction kinetics from the direct effect of SEI formation constitutes a major challenge. In contrast, there are numerous WISE examples that are not SEI-forming,^22–24^ still they offer notably extended stability voltage window.^7,10^ For instance, LiNO_3_-based WISE demonstrate a stability window of ≈2.55 V; it was revealed that decreased water activity plays only a minor role in this extension, it was suggested that kinetic effects are largely responsible.^25^ More recently, however it was shown that in LiNO_3_ electrolytes, a concurrent NO_3_^–^ complicates quantifying the respective influences of bulk electrolyte and interfacial structure on HER kinetics in WISE.^21^

In this work, we explore the origin of the extended stability window in WISE with a focus on how the local environment of water affects the kinetics and mechanism of the HER on platinum (Pt). We selected WISE based on ultrapure NaClO_4_ as a model system because it is non-SEI-forming and has lower viscosity compared to other WISEs, thus minimizing viscosity-related mass transfer effects (e.g., NaClO_4_ is significantly less viscous than acetate-based WISEs).^7,10,23^ We study HER kinetics on Pt, given its high HER activity and resistance to passivation;^7,26^ it is ideal for isolating the impact of local electrolyte structure on HER. We correlate HER activity with electrolyte concentration and structure using Fourier transform infrared (FTIR) spectroscopy and MD simulations. By analyzing bulk and interfacial structural changes within the electrolyte, we reveal their implications for HER kinetics.

## Results and Discussion

### Hydrogen Bonding, Electrolyte Structure, and Properties

NaClO_4_ in water can reach a concentration of 17 m, thus fulfilling water-in-salt conditions with 33 wt % of water and reaching a water-to-cation ratio as low as ∼3.2. To understand how this can impact HER kinetics, we first evaluated the influence of the NaClO_4_ concentration on the connectivity between water molecules by studying the series of electrolytes with different concentrations from dilute to WISE: 1, 5, 10, and 17 m NaClO_4_.

FTIR spectra of the region corresponding to the main water molecule’s vibrations show clear disruption of H-bonding with increased concentration of NaClO_4_ (Figure 1a). The broad water vibration band (ν­(OH)) can be deconvoluted into 3 bands, representing water molecules in different environments. Figure 1a indicates a progressive rise in the fraction of “isolated” water molecules, evidenced by the increased intensity of the ∼3600 cm^–1^ band.^27–30^ In contrast, the band at 3250 cm^–1^, associated with water connected through 4 hydrogen bonds to neighboring water molecules (often denominated as “ice-like”),^27–30^ is dominant in dilute solutions but diminishes as salt concentration increases. This supports the water-isolating effect of NaClO_4_ WISE. At intermediate concentrations (5 m NaClO_4_), water molecules with 1–3 hydrogen bonds (band ∼3400 cm^–1^),^28,30^ such as those in the vicinity of cations or those with replacement of H bonds between water molecules by weaker H bonds with ClO_4_^–^, become dominant, leading to an asymmetric environment. These changes in the water structure at higher electrolyte concentration correlate with a proportional increase in the intensity of the perchlorate peak ∼1100 cm^–1^,^30^ and its shift to lower wavenumbers (∼1078 cm^–1^), indicating stronger cation-perchlorate interaction and ion-pair formation (Figure S1).^30^

**Figure 1:**
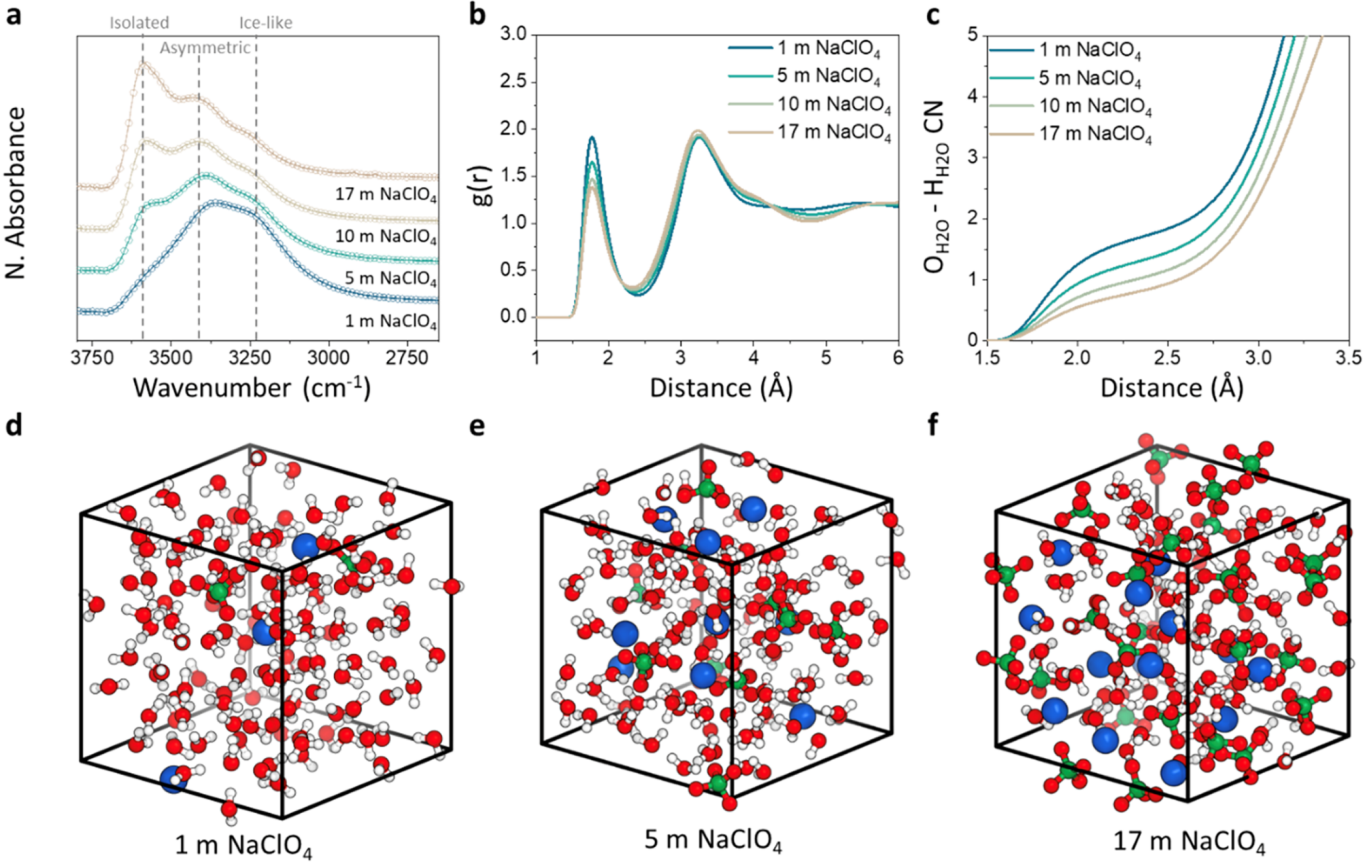
Electrolyte bulk structure at different concentrations. (a) FTIR spectra normalized by the water concentration. (b) MD generated RDF data *g*(*r*) for H_H_2_O_–O_H_2_O_ interactions. (c) MD generated average coordination number (CN) for the O_H_2_O_–H_H_2_O_ pair. (d–f) Zoom-ins of the MD simulation boxes showing the local ionic environments for the concentrations 1, 5, and 17 m NaClO_4_.

Next, to understand the changes of H-bonding and ionic environment at atomic scale, both classical MD simulations of the bulk electrolytes and constant potential MD simulations of a full electrochemical cell “Pt||*x* m NaClO_4_||Pt” at 1 V potential were carried out for *x* = 1, 5, 10, and 17 m (see Figure S2). The MD extracted bulk radial distribution functions (RDFs) (Figure 1b) show a gradual change in the water–water interactions as the salt concentration increases: while the location of the first peak at 1.76 Å stays the same, the first minimum, typically interpreted as the limiting distance of the first coordination shell, shifts to smaller distances, indicating a contraction of the water interconnected network as the salt concentration increases. Furthermore, the RDFs exhibit a reversal in the relative peak height of the two main peaks corresponding to the first and second coordination shells, respectively, showing the increasing displacement of water molecules in the local environment of water at higher concentrations.

RDFs of the ions’ main interactions show an increase in the ion-pair formation, as can be seen in Figure S3 and corresponding number integrals (NIs) in Figure S4, correlating well with the perchlorate band shift observed from the FTIR. The NIs for the water oxygen reveal the gradual change of the dominant coordinating atom from H_H2O_ to Na^+^ as the concentration increases (see Figures 1c and S4). Although at a salt concentration of 1 m, the water oxygen is coordinated by 1.7 H_H_2_O_ and 0.0 Na^+^ on average, at the highest concentration of 17.0 m, the coordination numbers shift to 0.7 and 1.0, respectively. Notably, the total number of neighbors for H_H_2_O_ and O_H_2_O_ does not change with concentration (see Figure S4). This is in agreement with the disruption of the H-bonding observed from FTIR and with literature results.^22,31^

The MD extracted structure factors *I*(*Q*) (Figure S5) also support the decrease in the amount of the free water with increasing concentration as evidenced by the decrease of water characteristic features at *Q* values between 2 and 3 Å^–1^, corresponding to the intermolecular H–O water first neighbor distance (see Figure 1b). The separation of water-rich and water-poor domains, as can be seen in the partial H_2_O–H_2_O *I*(*Q*), suggests the formation of nanosegregated clusters at *Q* < 1 Å^–1^ (Figure S5b). Qualitatively, this is also visible in the visualizations shown in Figure 1d–f, where water and cations separate from the anion-rich environment, corresponding to a “dissociating WIS” behavior.^9,25,32,33^

Next, we characterized the changes of physicochemical properties, such as conductivity, viscosity, and density of electrolytes with concentration, as summarized in Table 1. Notably, the viscosity of the 17 m solution is less than an order of magnitude higher than that of 1 m NaClO_4_. Walden analysis indicates that only 17 m solution can be classified as “good-ionic”, whereas electrolytes with lower concentrations (1, 5, 10 m) are moderately associated (Figure S6).^34,35^ Thus, because viscosity of the NaClO_4_ solutions increases marginally even when WIS condition is reached, while their structure and H-bonding network undergo dramatic changes, this solution series is an excellent model system to evaluate differences in HER kinetics between diluted and WIS electrolytes.

**Table 1:** Physicochemical Properties of the Electrolytes

concentration (molal)	conductivity (mS/cm)	viscosity (g cm^–1^ s^–1^)	density (g/cm^3^)	density (MD) (g/cm^3^)
1	75	0.010	1.08	1.07
5	180	0.015	1.31	1.32
10	164	0.030	1.50	1.55
17	117	0.074	1.67	1.75

### Kinetics of HER in WISE

Figure 2a shows that the HER overpotential (at 1 mA/cm^2^) increases almost linearly with rising salt concentration (and decreasing water concentration (Figure S7)). For 17 m NaClO_4_, WISE HER onset is delayed by 130 mV compared to 1 m electrolyte, shifting from −0.64 to −0.77 V vs SHE. Since, in unbuffered neutral electrolytes, such as NaClO_4_, HER takes place primarily by splitting of water molecules^36,37^ and the concentration of water decreases from 53.4 M for 1 m NaClO_4_ to 30 M for 17 m NaClO_4_. Therefore, next we investigated how water activity changes in WIS to estimate Nernstian shift.^38^ As can be seen from Figures 2b and S8, the measured water activity linearly decreases with salt concentration, dropping from 0.97 in 1 m solution to 0.47 for 17 m NaClO_4_. This is consistent with trends reported for other water-in-salt electrolytes^10^ and aligns with significant weakening of hydrogen bonding in WIS as observed from FTIR (Figure 1a). This water activity change could account for a Nernstian shift of about 20 mV between the most diluted and the most concentrated solutions. However, the experimentally observed shift is significantly larger (130 mV), suggesting significant contributions of other factors (discussed below).

**Figure 2:**
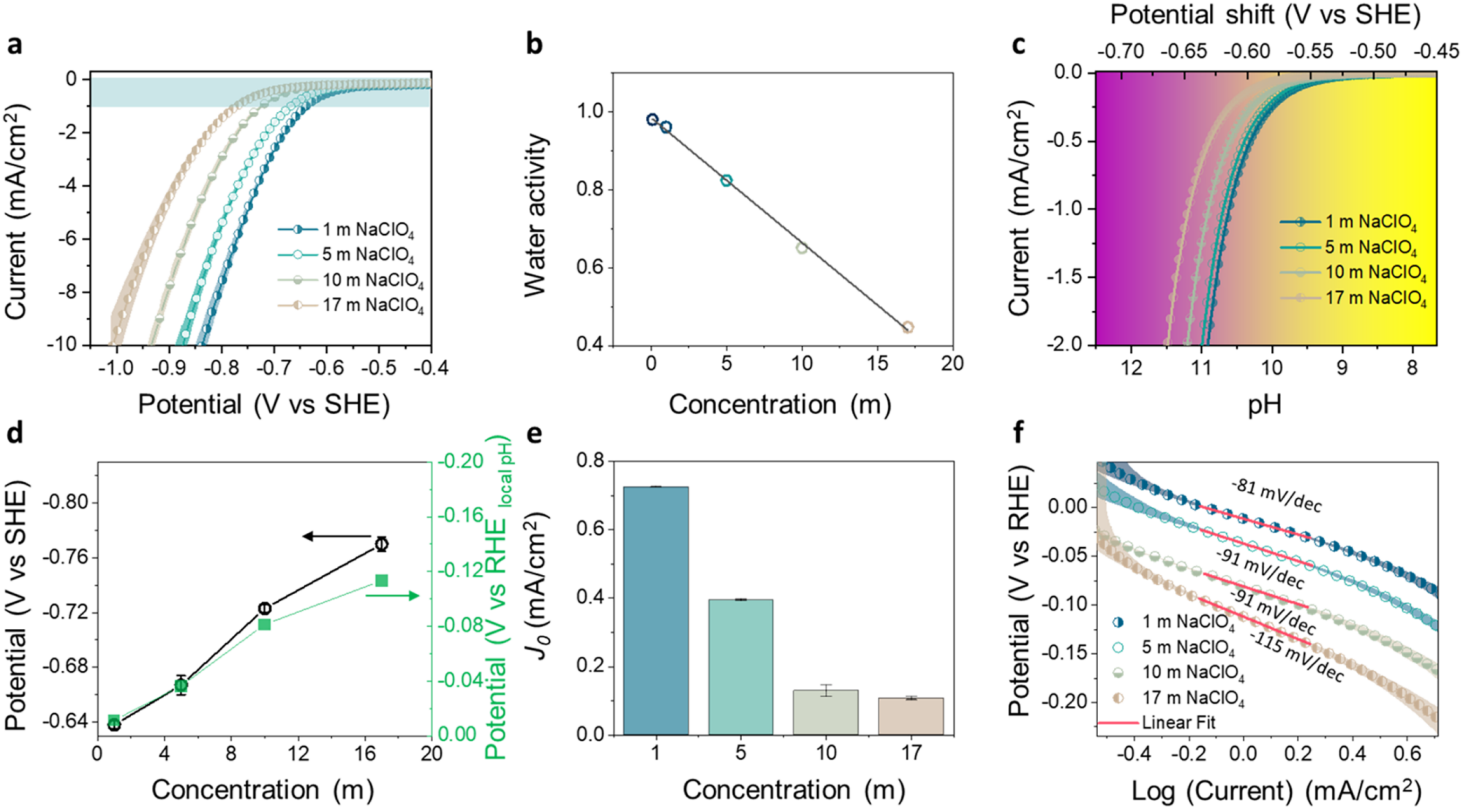
HER at a polycrystalline Pt electrode as a function of electrolyte concentration 1–17 m NaClO_4_. (a) LSV corrected by the electrochemical surface area (ECSA) collected in the negative scan direction at 50 mV/s in an RDE configuration at 1600 rpm for all electrolytes. (b) Measured water activity as a function of concentration. Activity was measured as the gas face equilibrium with the electrolyte. (c) Calculated local pH as a function of geometric current density for all electrolytes. (d) Overpotential required to reach 1 mA/cm^2^ as a function of concentration, bulk vs local pH. (e) Local pH-corrected exchange current as a function of concentration. (f) Local pH-corrected Tafel slopes generated from the LSV scans.

Next, given the unbuffered nature of the studied neutral solutions, we investigated the impact of local pH changes caused by proton consumption during hydrogen evolution on the HER onset potential. Conducting our experiments in RDE configuration with well-defined mass transfer conditions allowed us to calculate the local pH at the electrode surface as a function of the applied current, using the numerical approach that was introduced and validated by Auinger et al.^39^ The findings, presented in Figures S9a,b and 2c, reveal a substantial increase in local pH compared to the near-neutral bulk pH at a current density of 1 mA/cm^2^ (Table S2). Specifically, the local pH is estimated to rise to 10.6 for 1 m and up to 11.3 for 17 m WIS electrolytes (Figure S9). The calculated local pH changes in 1 and 17 m NaClO_4_ electrolytes were confirmed experimentally using the method by Liu et al.^40^ (see Figure S10). While these local pH changes lead to a Nernstian shift in the HER onset potential of up to 210 mV on the SHE scale (converted from the RHE scale, Figure S11), the contribution from concentration variations accounts for only ∼30 mV. Thus, upon accounting for the changes in the local pH as a function of concentration (Figure S11a,b), we reveal that the HER overpotential difference between 1 and 17 m NaClO_4_ is at ∼100 mV (Figures 2d and S11c,d), accounting for the variations in local pH for 1 m vs 17 m NaClO_4_. These findings indicate that the local pH at the electrode surface in nonbuffered concentrated neutral electrolytes is a significant factor contributing to delayed HER onset in WIS.

To further understand the influence of electrolyte concentration and structure on HER kinetics, we compared exchange current density values (***j*_0_**) for each electrolyte (Figure 2e) and Tafel slopes (Figure 2f) on the RHE scale, accounting for the local pH (Table S2).^36,37^ The *j*_0_ exhibits a 7-fold decrease when concentration is increased: from 0.72 mA/cm^2^ in 1 to 0.1 mA/cm^2^ in 17 m NaClO_4_. While Zhao et al. observed a similar magnitude decrease in *j*_0_ for impure LiTFSI WISE, the effect was primarily attributed to catalyst surface modification by Ag impurity deposition and SEI formation.^21^ In our case, since >99.99% pure NaClO_4_ salt is used, this reduction in exchange currents can be directly linked to the extended stability window of WIS electrolytes due to more sluggish HER kinetics as a result of modified interfacial electrolyte structure (discussed below).^25,41^ Similarly, the Tafel slopes increase with concentration from ∼81 mV/dec in 1 m electrolyte to ∼115 mV/dec in 17 m NaClO_4_ WIS, further supporting slower HER kinetics at higher concentrations. This trend can be rationalized by the shift in the rate-determining step mechanism of the HER from the Heyrovsky step (∼40 mV/dec, corresponding to second proton transfer) to the Volmer step (∼120 mV/dec, corresponding to first proton transfer).^36,42^ At lower concentrations (e.g., 81 mV/dec for 1 m NaClO_4_), the observed Tafel slopes fall between these expected values, aligning with previously reported values for similar electrolyte systems.^36,43^ These intermediate slopes can be attributed to the cation effect in alkaline electrolytes, where with Li^+^ a slope of 43 mV/dec is observed in contrast to 112 mV/dec with K^+^ on Pt(111) surfaces.^42^ On polycrystalline Pt, slopes of 70 mV/dec are observed for Li^+^ and Na^+^ cations, compared to 120 mV/dec for K^+^ and Cs^+^ cations,^43^ aligning well with our experimental results under diluted conditions.

We note that, because the electrolytes studied are unbuffered, the measured HER current reflects coupled kinetic and diffusion contributions.^39^ As such, the intrinsic exchange current density cannot be unambiguously determined, even after local pH correction,^39^ and the reported *j*_0_ values should be regarded as approximate. Nonetheless, the relative concentration-dependent trends remain meaningful and are further supported by the consistency between the values obtained for 17 m NaClO_4_ after local pH correction and those measured in 17 m NaClO_4_ adjusted to pH 13 with 0.18 m NaOH (Supplementary Discussion, Table S2, and Figure S12).

### Molecular Dynamics Simulations of Electrical Double Layer

To further understand the impact of electrolyte composition on HER kinetics, we analyzed changes in the electrical double layer speciation with rising NaClO_4_ concentration. To do so, we performed MD simulations of the polarized Pt(111)/electrolyte interface converged at a constant potential of 1 V (see Figure S13). We observed a change in the speciation of active sites on the Pt(111) surface as a function of concentration (Figure 3a–d): at low concentrations, the surface is covered almost entirely by an interconnected layer of water molecules adsorbed on the Pt(111) surface. This network is gradually disrupted as the concentration increases and the water coverage decreases. At a concentration of 5 m (Figure 3b), islands of interconnected and mobile water molecules are still present. At 10 and 17 m (Figure 3c,d), water molecules on the surface are strongly confined. Note that a mobile water molecule’s contribution may be dispersed over a wider area of the graph, whereas a highly constricted water molecule’s signal may be limited to a single adsorption site. These results are consistent with previous classical and ab initio MD studies performed on fluorinated WISE.^44^

**Figure 3:**
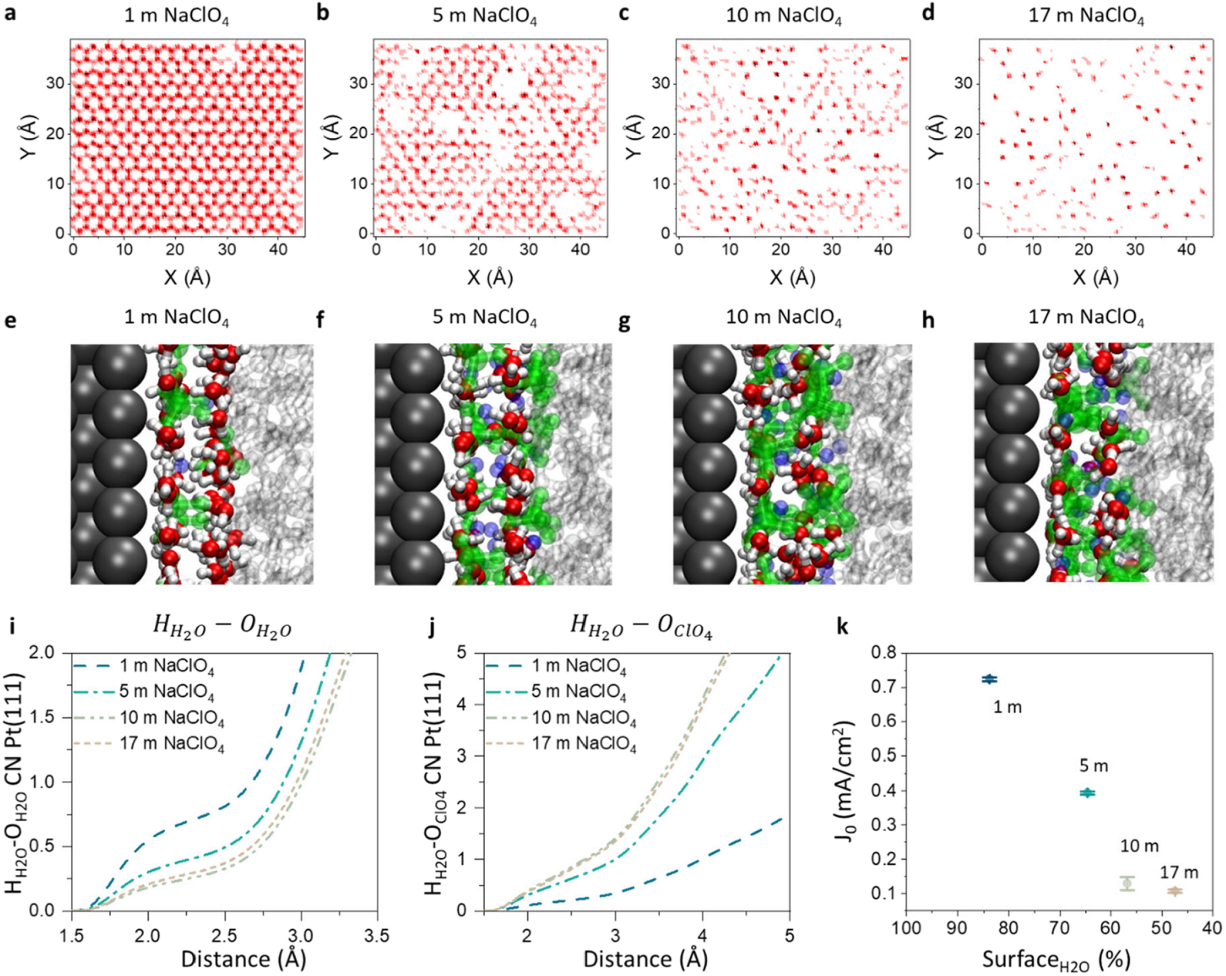
MD extracted surface coverage of the Pt(111) cathode surface by water and its correlation with HER kinetics. (a–d) Distribution function of water oxygen on the Pt(111) surface measured over 5 ns for 1, 5, 10, and 17 m NaClO_4_, respectively. (e–h) MD extracted Pt/electrolyte interface visualizations highlighting the first two layers of adsorbed ions and water. (i, j) Coordination numbers for the water interactions at the interface H_H_2_O_–O_H_2_O_, H_H_2_O_–O_ClO_4__, respectively. (k) Exchange current density vs the area covered by water *A*_H_2_O_.

Importantly, as the salt concentration increases from 1 to 17 m, the relative surface coverage of water molecules decreases from 85 to 44% (Figure S14). The molecular coverage at the inner and outer Helmholtz planes (first and second adsorption layers) shows a strong disruption of the water structure, as can be seen in Figure 3e–h. This induces changes in the H-bonding at the surface of the electrode, resulting in an altered H_2_O–H_2_O coordination number (see Figures 3i,j and S15). These observations were experimentally supported via in situ surface-enhanced FTIR spectroscopy (SEIRAS). The ν­(O–H) vibration region of interfacial water during HER on Pt shows an increased contribution of the isolated water band in 17 m NaClO_4_ relative to asymmetric and ice-like coordinated water, when comparing it to 1 m NaClO_4_ (see Figure S16).

When relating the changed water structure to the HER activity, we observe a strong correlation between water surface coverage and *j*_0_ with the concentration increase. Specifically, as the salt concentration rises from 1 to 17 m NaClO_4_, water surface coverage progressively decreases from 85 to 44% (Figure S14), corresponding with a significant reduction in *j*_0_ from 0.74 to 0.1 mA/cm^2^ (Figure 3k). While Zhao et al. observed a similar kinetic penalty in impure LiTFSI WISE, their effect was primarily attributed to catalyst surface modification by Ag impurity deposition and SEI formation.^21^ In our case, since >99.99% pure NaClO_4_ salt is used, this concentration-dependent decline in exchange current density suggests that HER activity is increasingly restricted to water-covered surface sites on the Pt catalyst.

Angular distribution functions (ADF) may provide further insight into the local structure at the electrode surface. ADFs of the angle between the O–H bonds of water molecules in the first adlayer on the cathode surface and the *z* axis (Figure S17) show a preferred angle of 96–104° corresponding to an orientation parallel to the surface, increasing the angle with concentration. At low salt concentrations, a secondary peak is present around 0° corresponding to an O–H bond pointing away from the surface. This orientation disappears at higher salt concentrations, indicating a reduction in hydrogen bonds between the first and second adlayers. A small but consistent number of water O–H bonds are found at an angle close to 180°, corresponding to a bond pointing toward the negatively polarized surface; at high salt concentrations, this peak translates into a shoulder at around 150°, corresponding to a tilted O–H bond pointing toward the surface.^45,46^ Performing the same analysis for simulations run at 0 V potential (Figure S17b) yields an increased occurrence of O–H bonds parallel to the surface and pointing away from the surface, whereas O–H bonds pointing toward the surface are absent.

### DFT Calculations and HER Mechanism

Our experimental data revealed an increase in the Tafel slope’s absolute value with the electrolyte concentration (Figure 2f), suggesting changes in the mechanism of the reaction.^36,47^ Changes in EDL speciation can alter the reaction mechanism by modulating local chemical environments, affecting individual energy barriers, and potentially changing the rate-determining step.^41,42,47–49^ Therefore, we next used density functional theory (DFT) to evaluate the energy barriers for each HER reaction step for different electrolyte concentrations. Specifically, we performed DFT calculations of the partial reactions on the Pt(111) surface to obtain activation barriers, using the nudged elastic band (NEB) approach.^50–52^ To analyze the influence on the reaction mechanism, we calculated and compared energy barriers for each HER reaction step expected in neutral and basic electrolytes.^36,42,47^ Volmer as the first electron exchange and Heyrovsky as the second electron exchange (see the solvation environments considered for the calculations in Figures S18 and S19):

$$\mathrm{Volmer},H_{2}O+e^{-}+Pt↔Pt‐H+{OH}^{-}$$

$$\mathrm{Heyrovsky},Pt‐H+H_{2}O+e^{-}↔H_{2}+{OH}^{-}+Pt$$

Figure 4a shows the energy barriers of the Volmer and Heyrovsky steps. The activation barrier for the Volmer step showed a decrease from 1.28 to 1.04 eV as the local environment of the reactants changes with concentration. Meanwhile, for the Heyrovsky step, a minor decrease of activation barrier from 1.26 to 1.17 eV is observed as the salt concentration increases. These trends of lower calculated barriers for the higher salt concentrations (in both forward and backward directions) do not follow the experimental trends of Tafel slopes (Figure 2f and Table S2) where the HER becomes more sluggish as the concentration increases, suggesting that even when there is a positive influence on the energy barriers from the ionic environment, a different limitation is ruling the changes in kinetics and mechanism, where at high concentrations, the reaction may be limited by the transport of active species to the surface.

**Figure 4:**
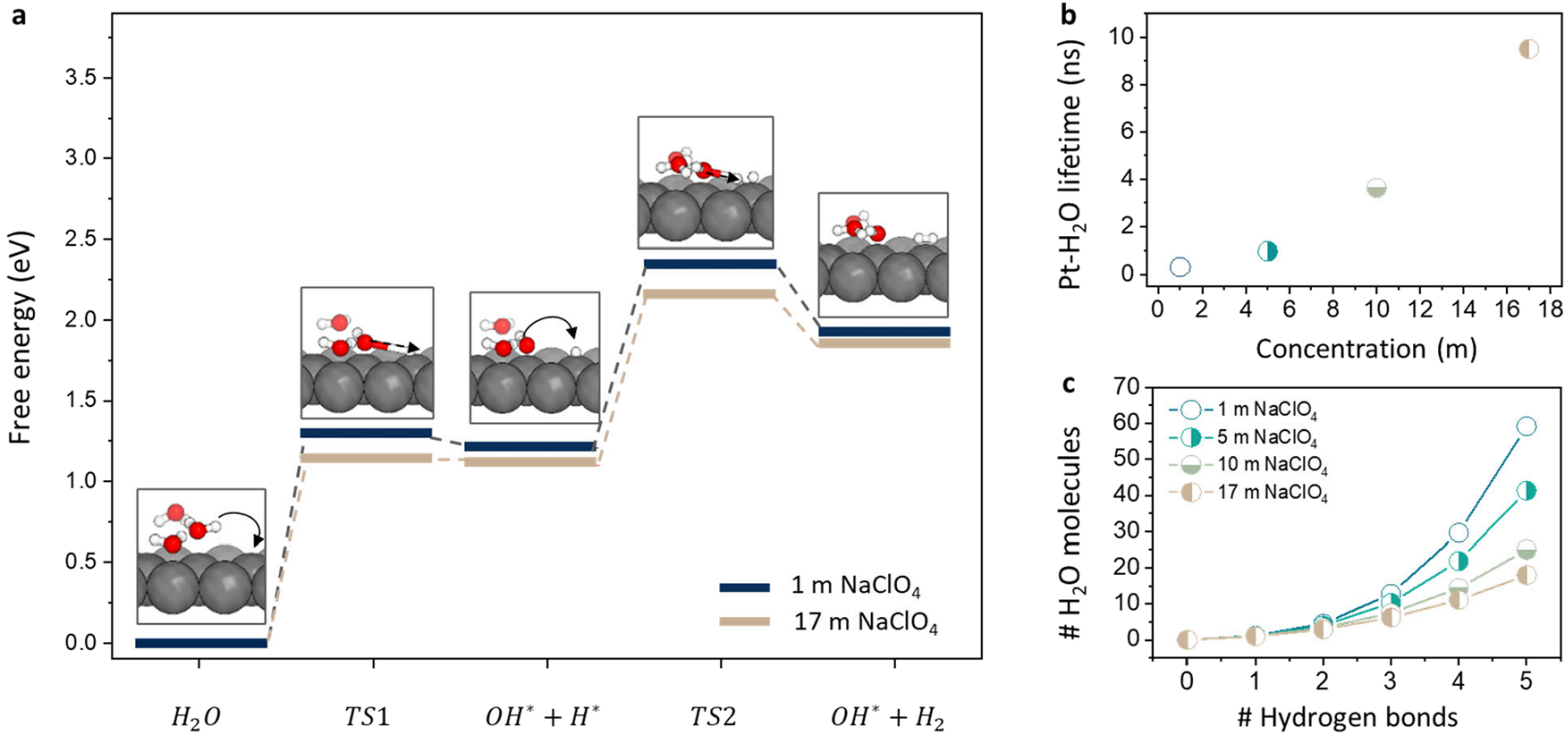
DFT calculations considered ionic environments at the interface. (a) Energy barriers for the reaction following a Volmer–Heyrovsky mechanism. Energies were calculated for an aqueous environment and high-salt-concentration environment. (b) MD extracted Pt–H_2_O intermittent interaction lifetimes calculated from pair existence autocorrelation functions. (c) MD extracted water percolation pathways for surface-adsorbed water molecules expressed as the number of interconnected water molecules vs the space extension of n-Hydrogen bonds.

To examine this in more detail, we analyzed the impact of the electrolyte concentration on the continuity of H-bonding networks near the electrode surface by studying percolation pathways, which may allow accelerated transport of OH^–^ anions through Grotthuss-like structure diffusion. Figures 4c and S20 show the average number of water molecules connected to water molecules adsorbed to the cathode surface as a function of the hydrogen bond chain length *n*, considering only hydrogen bonds that lead away from the surface. Althoug the network size is unaffected by the salt concentration at *n* = 1, i.e., the average number of hydrogen bonds per water molecule formed between water molecules in the first and second adlayer stays constant at 1.1, already at a chain length of *n* = 2, a decrease in the network size can be observed. As more extended hydrogen bond chains are considered, this decrease becomes more pronounced. Notably, the network size is more strongly affected as the concentration rises from 1 to 5 m compared to 10 to 17 m. These changes fully support the increased presence of OH^–^ and the lack of H_2_O at the inner Helmholtz plane at high concentrations. Furthermore, the average Pt–H_2_O interaction lifetimes drastically increase with rising salt concentration (Figure 4b), indicating a significant decrease in the mobility of surface-adsorbed species. Like the decrease in percolation pathways, this lower mobility may affect the local presence and transport of OH^–^.

## Conclusions

We isolate the influence of local electrolyte structure on the hydrogen evolution reaction (HER) kinetics on Pt across dilute to water-in-salt (WIS) concentrations of NaClO_4_. Our results show that the suppression of HER at high salt concentrations primarily stems from a local pH increase and sluggish water transport, while a reduced bulk water activity makes a relatively minor contribution. This occurs due to the disrupted hydrogen bonding in both the double layer and the bulk electrolyte, as our MD and IR analyses suggest. We observe that the exchange current depends almost linearly on the surface water coverage. As a result, the HER kinetics is slower: the exchange current density decreases by a factor of 7, and the Tafel slope increases 1.5 times when moving from dilute (1 m) to highly concentrated (17 m) regimes. Our findings show that the HER kinetics in WIS electrolytes depends on the local transport, interfacial water structure, and surface coverage (even on a highly active catalyst like Pt). We anticipate that these factors would be even more pronounced on less active catalysts.

## Supplementary Material



## Notes

Experimental methods: materials; electrolyte preparation; water activity; pH, conductivity, viscosity; FTIR (ATR) and SEIRAS; electrochemical protocols (cell configuration, RDE/RRDE, IR correction, potential conversion); electrode preconditioning and ECSA; local pH correction and measurement; Tafel slope analysis; MD and DFT (NEB) calculations; FTIR spectra and water-band analysis (Figure S1); MD cell snapshots (Figure S2); RDFs, coordination numbers, and structure factors (Figures S3–S5); Walden analysis (Figure S6); overpotential versus water concentration (Figure S7); water activity (Figure S8); computed local pH profiles and LSV zoom-ins (Figure S9); RRDE-based local pH measurements and collection-efficiency correction (Figure S10); HER LSV and Tafel slopes with/without local pH correction (Figure S11); alkaline control at pH 13 (Figure S12); interfacial density profiles, surface speciation, bilayer structure, and angular distributions (Figures S13–S17); NEB initial/final states for Volmer/Heyrovsky (Figures S18 and S19); hydrogen bond percolation analysis (Figure S20); SEIRAS setup and electrode characterization (Figure S21); cleaning/ECSA (Figure S22); bulk pH by indicator strips (Figure S23); bulk pH by meter and strips (Table S1); Tafel slope fits (bulk vs local pH) (Table S2); and MD system compositions and box dimensions (Table S3) (PDF)
