# Supplementary material for: Extended Stability Window in Water-in-Salt Electrolytes: Understanding the Origins
Source: J Am Chem Soc. 2025 Sep 18;147(39):35953–61. doi: 10.1021/jacs.5c12989 (PMC12498405; doi:10.1021/jacs.5c12989)
Supplement: Supplementary file 1 [file ja5c12989_si_001.pdf]

Supplementary information

Extended Stability Window in Water-in-Salt Electrolytes: Understanding the Origins

Dario Gomez Vazquez[a,ǂ], Johannes Ingenmey[b,d,ǂ], Katharina Trapp[a], Dennis Ciliak [a], Mathieu Salanne[b,c,*], Maria R. Lukatskaya[a,*]

[a] Department of Mechanical and Process Engineering, ETH Zurich, 8092 Zurich, Switzerland
[b] Sorbonne Université, CNRS, Physico-Chimie des Électrolytes et Nanosystèmes Interfaciaux, PHENIX, F-75005 Paris, France
[c] Institut Universitaire de France (IUF), 75231 Paris Cedex 05, France
[d] University of Bonn, Mulliken Center for Theoretical Chemistry, 53115 Bonn, Germany
[ǂ] these authors contributed equally
*Correspondence: mathieu.salanne@sorbonne-universite.fr and mlukatskaya@ethz.ch

TABLE OF CONTENTS

EXPERIMENTAL SECTION....3
- **Materials**....3
- **Electrolyte preparation**....3
- **Water activity**....3
- **pH and conductivity**....3
- **Electrolyte viscosity**....3
- **FTIR spectroscopy**....3
- **Electrochemical characterization**....3
- **Working electrode (WE) preconditioning and ECSA determination**....4
- **Local pH correction**....4
- **Local pH measurement**....5
- **Tafel slope analysis**....7
- **Classical molecular dynamics (MD) simulations**....7
- **Density functional theory (DFT) calculations**....8

SUPPLEMENTARY FIGURES....10
SUPPLEMENTARY TABLES....23
SUPPLEMENTARY REFERENCES....26

EXPERIMENTAL SECTION

Materials

Sodium perchlorate hydrate (>99.99%) was purchased from Sigma Aldrich. The salt was dried overnight under vacuum at 150°C to remove water. After drying, the salts were used immediately to prepare the electrolyte solutions.

Electrolyte preparation

A concentrated electrolyte (17 m) was prepared using milliQ water, all other electrolytes were prepared by diluting the most concentrated solution.

Water activity

Water activity was measured using a water activity meter LabMaster-aw neo (Novasina). The instrument was calibrated beforehand using water activity standards provided by Novasina.

pH and conductivity

The conductivity of the electrolytes were measured using an Oakton PC 2700 benchtop instrument. The pH of the electrolyte was measured at least 3 times with the InLab® Max Pro-ISM pH sensor (Mettler Toledo). Measurements were taken at 25°C. Both sensors were calibrated before each set of measurements using calibration standards from Oakton. Here we note that pH measurements of solutions with high salt concentration, when performed with a glass pH probe, can be influenced by non-zero liquid-junction potentials; therefore, their absolute values cannot be claimed with high accuracy. Consequently, the pH values presented in **Table S1** should be regarded as an indication of the near-neutral pH of these solutions. In addition, we measured pH using pH indicator strips (Macherey-Nagel GmbH & Co. KG), a method recommended by Boettcher et al.[1] for highly concentrated electrolytes, as shown in **Figure S23.**

Electrolyte viscosity

Viscosity of the solutions were measured using an oscillatory rheometer (ARES-G2, TA Instruments). The measurements were performed in the cone-plate geometry. The shear rate varied from 100 to 1000 Hz.

FTIR spectroscopy

The FTIR spectra of the electrolytes were measured in ATR configuration using a Nicolet is50 FTIR instrument equipped with a Veemax III ATR unit and a Si wafer (IRUBIS) as ATR element. Each spectrum consists of 128 co-added scans and was acquired with a resolution of 4 cm^{-1} using a D-LaTGS detector. SEIRAS measurements were conducted int the same setup utilizing a home-build cell (**Figure S21a**) consisting of a leakless Ag/AgCl reference electrode, Pt coil counter electrode and Pt-thin-film working electrode (7 mm diameter) coated on a Si wafer (IRUBIS, specialized 1). The Pt-thin film electrode was prepared in a two-step process as published previously.[3] First, a gold thin-film was prepared using an electroless deposition method, then Pt was deposited through electrochemical deposition (Chronopotentiometry, -144 µA, 6 min), using 4 mM H_2PtCl_6 in 0.7 M NaH_2PO_4.[3] The CV characterization of the prepared SEIRAS-active Pt thin-films as well as the HER current response during SEIRAS measurements were included into **Figure S21c and d.** The spectra were collected utilizing an MCT detector and a resolution of 8 cm^{-1}. 24 scans were coadded to achieve a time resolution of 10s.

Electrochemical characterization

All electrochemical measurements were conducted using a VSP-300 Biologic® potentiostat. A two-compartment cell was used to separate the working electrode from the reference and counter electrodes, employing an ion-selective Nafion membrane. All electrolytes were purged from O_2 by purging with N_2 for 15 minutes prior to the measurement and in between experiments. During electrochemical measurements back flow of O_2 was avoided by actively purging the cell headspace with N_2. The working electrode (WE) was a

polycrystalline Pt disc (4 mm diameter). An Ag/AgCl electrode in 1 M KCl (CH instruments) was used as the reference electrode. A platinum (Pt) coil served as the counter electrode. Measurements were performed with a rotating disk electrode (RDE) apparatus (ALS-3A) at 1600 rpm. The hydrogen evolution reaction (HER) was studied by means of linear sweep voltammetry (LSV) at 50 mV/s. Electrochemical impedance spectroscopy was measured with a capacitive shunt on the reference electrode.[2] The IR correction was initially performed to 85% by the instrument, and further adjusted manually to achieve full 100% correction, enabling accurate comparisons across varying electrolyte concentrations. All potentials were converted from Ag/AgCl to either the standard hydrogen electrode (SHE) or reversible hydrogen electrode (RHE) using the following equations:

$$E_{SHE} = E_{Ag/AgCl} + 0.223$$

$$E_{RHE} = E_{Ag/AgCl} + 0.059 * pH_{Bulk/local} + 0.223$$

In case bulk pH was used for the calculation, it was assumed to be 7.

Working electrode (WE) preconditioning and ECSA determination

The Pt WE was mirror-polished with 300 nm alumina powder, washed, and sonicated twice in deionized (DI) water to remove any residual alumina. Electrochemical preconditioning was performed by cycling from 0 to 1.23 V vs. RHE at 1000 mV/s for 100 cycles in 100 mM $HClO_4$. After preconditioning, the electrochemical surface area (ECSA) was determined from hydrogen underpotential deposition region current (H-UPD) by cycling the electrode at 50 mV/s from 0.06 to 0.6 V *vs.* RHE in the same electrolyte (**Figure S22**).[3]

Local pH correction

To estimate for the local pH at the electrode surface, we applied equations derived by Auinger et al.[4], which are specifically derived for the case of non-buffered electrolytes. These equations are valid for reactions where H^+ or OH^- ions are directly involved, under the assumption that the only process occurring is faradaic with 100% coulombic efficiency towards the desired reactions. For the hydrogen evolution reaction (HER) under neutral conditions, the primary reaction is:

$$2H_2O + 2e^- \leftrightarrow H_2 + 2OH^-$$

Due to the 1:1 stoichiometric correlation between electrons and OH^- produced (or H^+ consumed in acidic conditions), the current can be directly correlated with the H^+/OH^- concentration at the electrode interface. Assuming constant mass transport with no accumulation, this relationship can be expressed as:

$$J_{H^+/OH^-} = \frac{F}{\delta_{eff}}\left[D_{H^+}\left(C_{H^+}^{surf} - C_{H^+}^{bulk}\right) - D_{OH^-}K_W\left(\frac{1}{C_{H^+}^{surf}} - \frac{1}{C_{H^+}^{bulk}}\right)\right]$$

Where:

- δ_{eff} is the effective diffusion layer thickness,
- F is the faraday constant,
- D_{H^+} and D_{OH^-} are the diffusion coefficients for H^+ and OH^- respectively.
- $C_{H^+}^{surf}$ and $C_{H^+}^{bulk}$ are the proton concentrations at the electrode surface and in the bulk electrolyte, respectively,
- K_W is the equilibrium constant for water dissociation.

The effective diffusion layer thickness (δ_{eff}) is a function of the individual diffusion layers and the concentrations of H^+ and OH^-:

$$\delta_{eff} = \frac{\delta_{H^+}C_{H^+} + \delta_{OH^-}C_{OH^-}}{C_{H^+} + C_{OH^-}}$$

The individual diffusion layer thickness (δ_i) is calculated using the Levich equation:

$$\delta_i = 1.6126 D_i^{\frac{1}{3}} v^{\frac{1}{6}} \omega^{-\frac{1}{2}}$$

Where:

- D_i is the diffusion coefficient of the specie "i" in m^2/s,
- v is the kinematic viscosity in m^2/s, and
- ω is the rotation rate in s^{-1}.

For the $NaClO_4$ electrolytes used in our study, changes in concentration are accompanied by changes in electrolyte viscosity. Since the diffusion layer thickness is dependent on viscosity, and the diffusion coefficients are inversely proportional to the dynamic viscosity, it is necessary to account for viscosity variations in the system. The diffusion coefficients were estimated using the Stokes-Einstein-Sutherland equation: [5]

$$D = \frac{k_b T}{6\pi\eta r}$$

Where:

- k_b is the Boltzmann constant,
- T is the temperature in Kelvin,
- η is the dynamic viscosity and
- r is the hydrodynamic radius.

We employed literature values for the diffusion coefficients of H^+ and OH^- in water,[4] adjusting them using experimentally determined viscosity values.

To determine the real open circuit potential (OCP) for HER and compensate for the visible onset potential, the relationship between potential and local pH was derived from the Nernst equation:

$$E = E^0_{H_2/H^+} \frac{RT}{2F} \ln\left(\frac{a^2_{H^+}}{a_{H_2}}\right) \approx -59\, mV * pH$$

Assuming the H_2 concentration remains constant, the potential depends solely on the local pH.

The resulting local pH gradients as a function of geometric current density are shown in **Figure S9**. These gradients were used to correct the potential vs. RHE using a Python-based interpolation script. The script matches the experimentally measured geometric current values to their corresponding local pH and calculates the corrected potential vs. RHE. Subsequently, the current values were adjusted to compensate for the electrochemically active surface area (ECSA), as illustrated in **Figure S22** and summarized in **Table S3**.

Local pH measurement

The local pH measurements were conducted using the rotating ring-disk electrode (RRDE) with Pt disk and gold (Au) ring. The RRDE dimensions were 0.2 cm, 0.25 cm, and 0.35 cm for the disk radius (r_1), inner ring radius (r_2), and outer ring radius (r_3), respectively. The used pH detection method was adapted from Liu et al.[6]

Preparation of the modified Au ring. The surface of the Au ring was modified with 4-nitrothiophenol (4-NTP, Merck, 80%) by immersion in a solution of 1 mM 4-NTP in ethanol for approximately 20 min. After rinsing the RRDE thoroughly with ethanol and water, the 4-NTP on the ring was electrochemically reduced to 4-hydroxylaminothiophenol (4-HATP) in 0.1 M H_2SO_4 using cyclic voltammetry (four scans at 200 mV/s between 0.08 and 0.64 V vs. RHE). The modified Au ring acts as a pH sensor based on the redox activity of the 4-HATP/4-nitrosothiophenol (4-NSTP) couple. The potential of the redox peak, defined as the midpoint between the anodic and cathodic peaks, shifts with pH and can thus be used to monitor local pH.

Measurements in 1 m and 17 m $NaClO_4$ were conducted in a beaker cell with the RRDE connected in bipotentiostatic mode. The setup included two working electrodes (disk and ring), an Ag/AgCl reference electrode (CH Instruments Inc., 1 M KCl), and a Pt coil counter electrode. Prior to measurements, the system was calibrated in a series of 1 m $NaClO_4$ solutions with pH values of 7.1, 9.2, 9.7, 10.9, and 12, measured using an InLab® Max Pro-ISM pH sensor (Mettler Toledo). During calibration, the disk was held at open-circuit potential while the ring was cycled at 200 mV/s within a 0.4 V potential window ranging from 0.1 to −0.6 V vs. Ag/AgCl (see **Figure S10 a**). Redox peak potentials were extracted from the cyclic voltammograms (CVs), and a linear calibration curve was obtained (see **Figure S10 b**).

After calibration, chronopotentiometry was carried out at the disk using a sequence of current density steps (−0.01, −0.1, −0.2, −0.5, −1.0, −1.4, and −1.8 mA/cm^2), each applied for at least 1 minute. Simultaneously, the ring was cycled at 200 mV/s within a 0.4 V window between 0.0 and −0.5 V vs. Ag/AgCl (**Figure S10 c-d**). Similarly to calibration, the redox peak potentials were extracted from the ring voltammograms and converted to local pH values using the parameters from the linear fit of the calibration curve:

$$pH_{ring} = \frac{(E_{redox} - B)}{A}$$

Where:

- A is the slope of the linear fit.
- B is the y-intercept of the linear fit.
- E_{redox} is the redox peak potential.

Consequently, the ring pH can be converted into the local pH at the disk with the equations below:

$$pH_{disk} = -log_{10}\left(\frac{f_{pH} + \sqrt{{f_{pH}}^2 + 4 \cdot K_W}}{2}\right)$$

With:

$$f_{pH} = \frac{1}{N_D}\left(c_{ring,H^+} - c_{ring,OH^-}\right) - \frac{(1 - N_D)}{N_D}\left(c_{bulk,H^+} - c_{bulk,OH^-}\right)$$

Where:

- K_W is the equilibrium constant for water dissociation.
- c_{ring,H^+} and c_{bulk,H^+} are the proton concentration on the ring and in the bulk, respectively.
- c_{ring,OH^-} and c_{bulk,OH^-} are the hydroxide concentration on the ring and in the bulk, respectively.
- N_D is the collection.

The resulting local pH gradients in 1 and 17 m $NaClO_4$ as a function of geometric current density are shown in **Figure S10 e-f**. These exhibit similar trends and shifts with concentration when compared to the calculated local pH gradients. Although the measured pH values are approximately 0.5 units lower than calculated, they support the conclusions regarding HER overpotential differences between 1 m and 17 m $NaClO_4$. This consistent offset is likely due to the radial pH gradient generated by HER at the disk extending over the ring. As a result, the inner ring region, being closer to the disk, experiences a higher local pH than the outer ring region.

Since the redox peaks reflect the cumulative pH response by the 4-HATP/4-NSTP couples across the Au ring surface, the observed peak position is broadened and shifted. As the radial distance from the disk increases, the encompassed area of the ring increases as well. Accordingly, the outer region of the ring can accommodate more surface adsorbed molecules than the inner region and thus contributes more significantly to the overall

redox peak position. Moreover, the pH gradient flattens toward the outer edge of the ring, meaning that successive increments in radial distance correspond to molecules experiencing very similar pH environments. This results in redox currents occurring at similar potentials, further shaping the observed peak.

Consequently, the redox peak is biased towards lower pH values, underestimating the pH at the disk when the collection efficiency (42.2%) for the present RRDE geometry is used. To correct for this radial bias, we propose calculating the collection efficiency based on the outermost 10% of the ring radius. This approach offers a reasonable compromise between capturing the dominant contribution from a flatter pH gradient at the outer ring region and ensuring a sufficiently large area to generate a measurable current. Applying the equations below proposed by Albery et al.[7] and the adjusted RRDE geometry for the last 10% of the ring radius (r_1 = 0.2 cm, r_2 = 0.34 cm, and r_3 = 0.35 cm), the collection efficiency (N_D) comes to 8.5%. This correction aligns the measured pH with the calculated pH values (see **Figure S10 e-f**).

$$N_D = 1 - F\left(\frac{\alpha}{\beta}\right) + \beta^{\frac{2}{3}}[1 - F(\alpha)] - (1 + \alpha + \beta)^{\frac{2}{3}}\left\{1 - F\left[\left(\frac{\alpha}{\beta}\right)(1 + \alpha + \beta)\right]\right\}$$

With:

$$F(\theta) = \frac{\sqrt{3}}{4\pi}\ln\left[\frac{\left(1 + \theta^{\frac{1}{3}}\right)^3}{1 + \theta}\right] + \frac{3}{2\pi}\arctan\left(\frac{2\theta^{\frac{1}{3}} - 1}{\sqrt{3}}\right) + \frac{1}{4} \qquad \alpha = \left(\frac{r_2}{r_1}\right)^3 - 1 \qquad \beta = \left(\frac{r_3}{r_1}\right)^3 - \left(\frac{r_2}{r_1}\right)^3$$

Where:

- r_1 is the disk radius.
- r_2 is the inner ring radius.
- r_3 is the outer ring radius.

Tafel slope analysis

Tafel slopes were determined for potentials referenced to the RHE scale, considering either the bulk pH or the local pH from the numerical model (See **Figure S11, Figure S12** and **Table S2**). To ensure reproducibility, 5–8 independent experiments were conducted for each solution. The values reported in this study represent the average results, with confidence intervals calculated from the standard deviation of the experimental data.

Additionally, we performed a measurement in 17 m $NaClO_4$ solution adjusted to pH 13 with 0.1 M NaOH (0.18 m NaOH). At such high bulk pH, the local pH can be assumed to match the bulk pH, as the amount of OH^- generated at current densities between 0 and −10 mA/cm^2 would increase the local pH by less than 0.01 pH units from pH 13. Under these strongly basic conditions, an overpotential of 0.090 V vs RHE is required to reach 1 mA/cm^2, while the exchange current density (***j_0***) reaches the same value of 0.107 mA/cm^2 as during the measurements in neutral 17 m $NaClO_4$ (see **Table S2** and **Figure S12**). These results indicate that local pH is a significant factor contributing to the extension of the voltage stability window in neutral electrolytes when using polycrystalline Pt as the catalyst. Moreover, the lower Tafel slope of 75 mV/dec at bulk pH of 13 compared to the neutral pH measurements suggests that WIS are less effective at suppressing HER under strong alkaline conditions, likely due to the faster transport of OH^- relative to water in neutral pH systems and the difference in the initial state of the electrode.

Classical molecular dynamics (MD) simulations

MD simulations were performed using the LAMMPS program package (version 23 Jun 2022).[8] The composition and final box dimensions of the simulated systems are given in **Table S3**. OPLS-AA[9] and OPLS-2009IL[10] force field parameters were employed for ClO_4 and Na, respectively, and the SPC/E force field[11] was used for water.

Lorentz-Berthelot mixing rules were applied for non-bonded interactions between unlike atoms.[12] The cutoff for Lennard-Jones and Coulombic interactions was set to 1.2 nm. Initial configurations were created randomly using the PACKMOL tool.[13]

Bulk simulations of all concentrations were carried out in cubic boxes, employing periodic boundary conditions in all directions. The systems were equilibrated over a total simulation time of 4.7 ns in the NVE, NpT, and NVT ensemble. After an initial energy minimization, the system was simulated for 0.03 ns in the NVE ensemble with added velocity scaling corresponding to a temperature of 500 K. Afterwards, the systems were simulated for 3.8 ns in the NpT ensemble. The Nosé-Hoover chain thermostat and barostat were applied to achieve a constant pressure of 1.01325 bar and a temperature of 298.15 K.[14, 15] The average volume of the latter 3 ns was then used as final box volume for the subsequent equilibration of 0.9 ns in the NVT ensemble. Finally, a production run of 5 ns was carried out in the NVT ensemble. The timestep was set to 0.5 fs and every 1000th step was saved in a trajectory for further processing.

Simulations of the electrified solid-liquid interface

We used the ELECTRODE package[16] for constant potential simulations implemented in LAMMPS. Periodic boundary conditions were applied in x and y direction. In the z direction, the systems were confined by a 16x16x5 Pt surface slab on both sides of the simulation box, which acted as electrodes during the constant potential simulations. The 12—6 Lennard-Jones force field parameters for face-centered cubic metals by Heinz et al. was used to model non-Coulombic interactions with Pt atoms.[17] A lattice constant of 3.92 Å was used to construct both the Pt(100) and Pt(111) electrodes and the Pt atoms were frozen during the entire simulation. The distance between both electrodes was set such that the confined liquid phase reproduces the bulk density. A buffer of 2.5 Å was added to account for the gap between solid and liquid phase. The systems were equilibrated for a total simulation time of 3.7 ns. For the first part of the equilibration, the atom charge of all Pt atoms was set to 0. After an initial energy minimization, the systems were simulated for 0.03 ns in the NVE ensemble with added velocity scaling corresponding to a temperature of 500 K. The systems were then simulated for 1.7 ns in the NVT ensemble at a temperature of 298.15 K. Afterwards, the constant potential method was applied to set the atomic charges of the Pt electrodes such that a potential of 1 V is achieved. The systems were then allowed to equilibrate for 2 ns under the constant potential. Finally, a production run of 10 ns was carried out under the same settings. The timestep was set to 1 fs and every 1000th step was saved in a trajectory for further processing.

Radial distribution functions (RDF), Pt-H_2O interaction lifetimes, and structure factors (as implemented in Ref.[18]) were calculated from the MD simulations using the TRAVIS code for trajectory analysis.[19, 20] The lifetimes were obtained from calculating intermittent pair existence autocorrelation functions of surface Pt atoms and water molecules. The relative surface occupation of each component was estimated by performing a two-dimensional radical Voronoi tessellation of all atoms in the first adsorption layer, using Bondi's list of van der Waals radii[21] and assuming full surface coverage.

Percolation pathways were calculated by defining a group of reference water molecules (e.g., molecules present in the first adsorption layer on the electrode surface) and counting the number of water molecules connected to the reference molecule through a number of n hydrogen bonds (with $d(O\cdots O) \leq 3.4$ Å and $\angle(O\text{-}H\cdots O) \geq 120°$). In any hydrogen bond chain, each water molecule was counted only once, so as to avoid counting closed rings of hydrogen bonds. The obtained numbers were averaged over the entire MD trajectory and the number of reference molecules. Additional criteria were applied to differentiate percolation pathways parallel to the electrode surface (considering only hydrogen bonds between surface-adsorbed water molecules) and perpendicular to the electrode surface (excluding hydrogen bonds between surface-adsobred water molecules). The analyses were carried out using custom Python 3 scripts.

Density functional theory (DFT) calculations

DFT were performed using the CP2K quantum chemistry software package.[22] To obtain the activation barriers for the various partial reactions involved in the HER mechanism, nudged elastic band (NEB) calculations were carried out.[23-25] In all calculations the PBE functional was used in combination with the molecularly optimized

double-ζ basis set (MOLOPT-DZVP-SR-GTH) and the corresponding PBE Goedecker–Teter–Hutter pseudopotentials for core electrons.[26-28] The D3 dispersion correction was applied to account for London interactions.[29, 30] The Pt(111) surface was modeled by a 6x6x4 surface slab, using the same lattice constant of 3.92 Å. The atomic positions of the surface slab were kept fixed throughout all calculation Periodic boundary conditions were applied in all directions with a box size of approximately 16.6 Å x 14.4 Å x 25.0 Å. All NEB calculations were carried out with no explicit solvent molecules, with two water molecules, and with one water molecule paired with a $NaClO_4$ ion pair to model the conditions in vacuum, at low salt concentrations, and at high concentrations, respectively. Initially, the isolated molecules and fragments were optimized on the Pt(111) to verify their preferred adsorption sites. The initial and final atomic configurations for the NEB calculations were then constructed by placing the reactants (or products) in close proximity to each other on their preferred adsorption sites. The position of the explicit solvent molecules were determined using the ABCluster code[31] at classical force field level. The initial and final configuration were then optimized at DFT level. Finally, a number of 14 intermediate steps between initial and final state were optimized with the NEB method.

SUPPLEMENTARY FIGURES

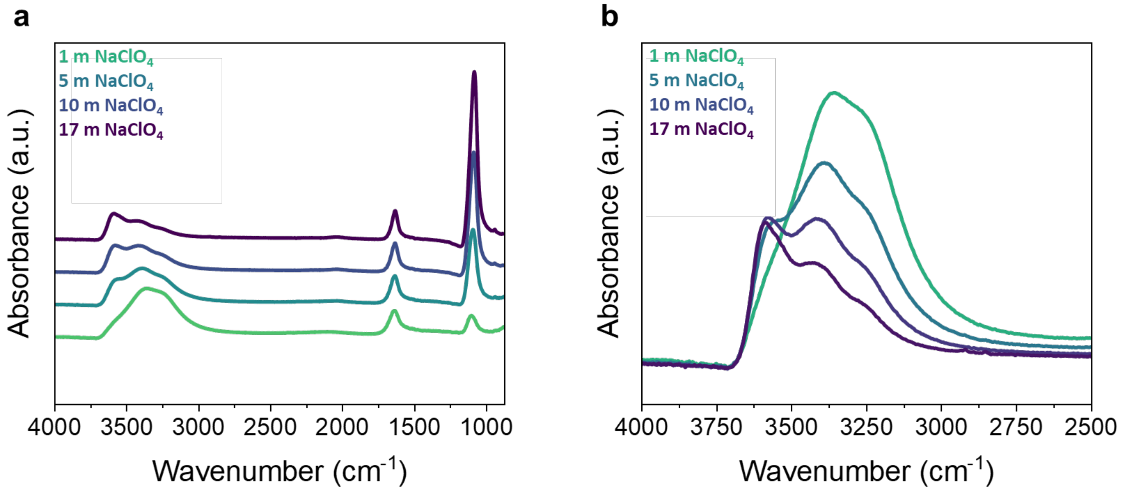

Figure S1. FTIR spectra collected in ATR configuration. a) shows the full spectra. b) shows the water band region from 2750 to 3750 cm^{-1}.

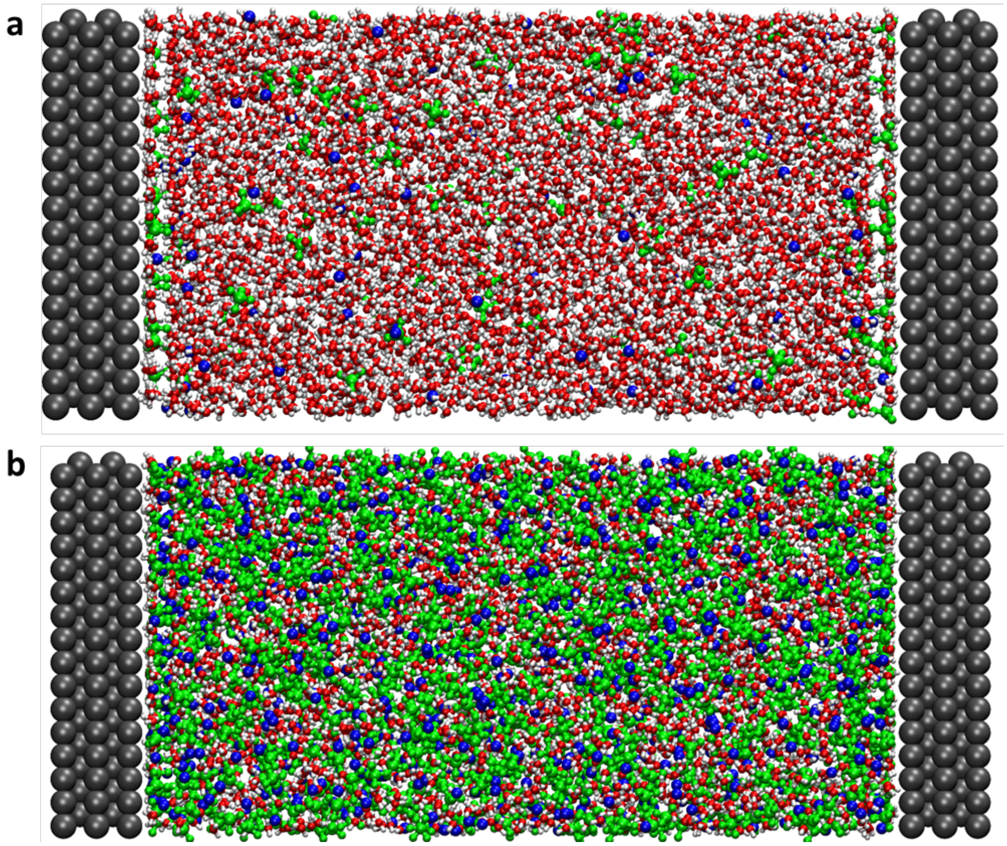

Figure S2. MD simulation box visualizations. a shows the simulation box for 1 m $NaClO_4$ with Pt(111) electrodes and b) for 17 m $NaClO_4$.

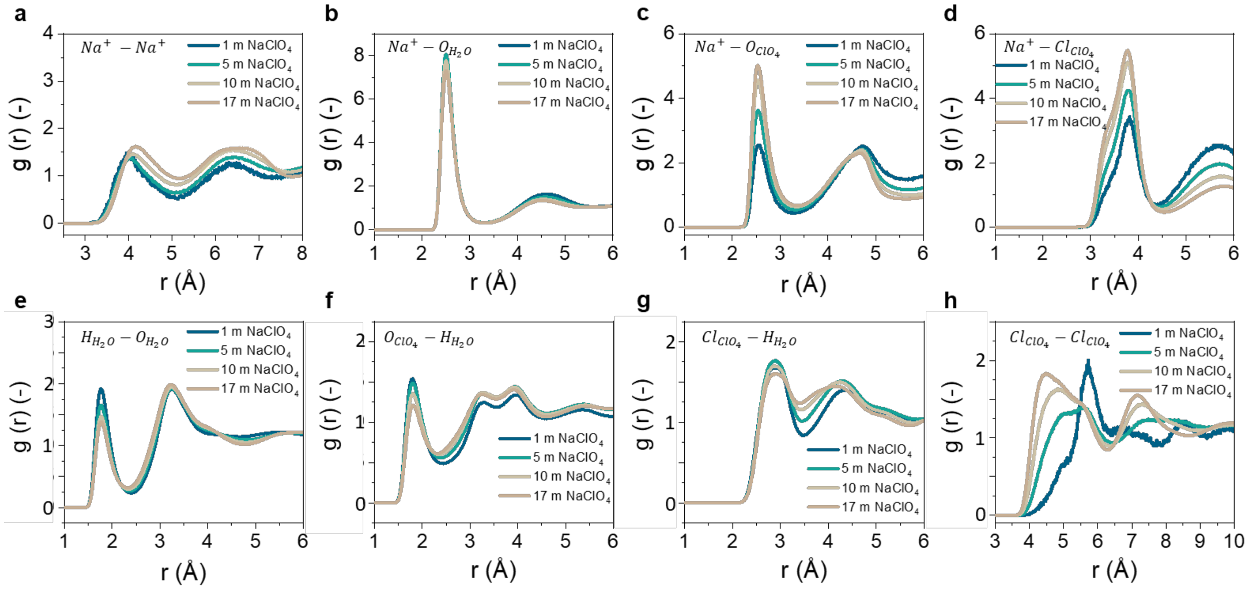

Figure S3. MD generated RDF in g(r) space for the main intermolecular interactions as a function of concentration. a) $Na^+ - Na^+$ b) $Na^+ - O_{H_2O}$ c) $Na^+ - O_{ClO_4}$ d) $Na^+ - Cl_{ClO_4}$ e) $H_{H_2O} - O_{H_2O}$ f) $O_{ClO_4} - H_{H_2O}$ g) $Cl_{ClO_4} - H_{H_2O}$ h) $Cl_{ClO_4} - Cl_{ClO_4}$.

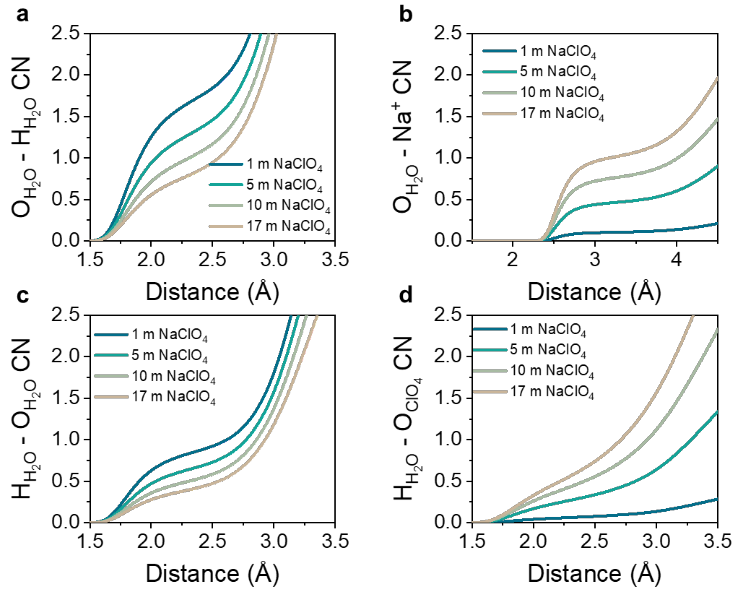

Figure S4. MD generated Coordination number for main water interactions. a) $O_{H_2O} - H_{H_2O}$ b) $O_{H_2O} - Na^+$ c) $H_{H_2O} - O_{H_2O}$ d) $H_{H_2O} - O_{ClO_4}$

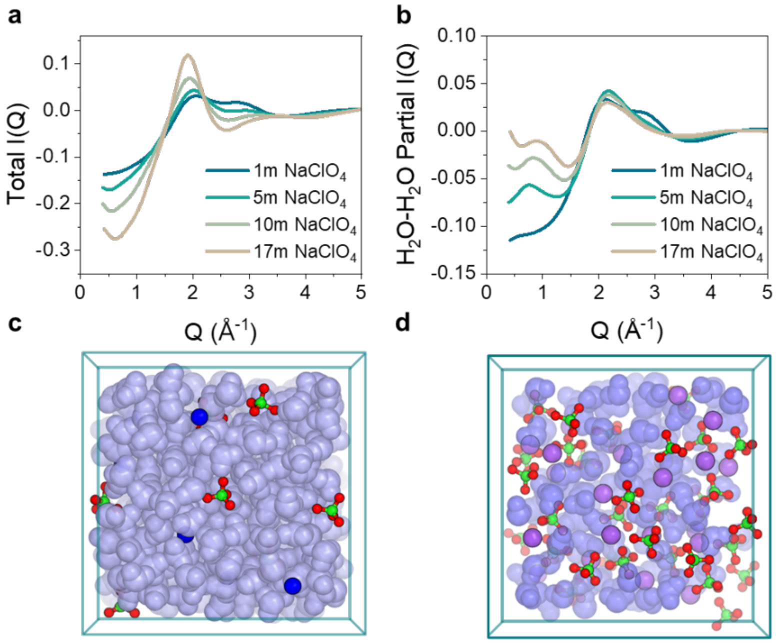

Figure S5. MD generated scattering I(Q) for the electrolyte at different concentrations and its respective visualizations. a) Total scattering b) Partial scattering $H_2O - H_2O$ c) MD Zoom in visualization 1 m $NaClO_4$ d) MD Zoom in visualization 17 m $NaClO_4$

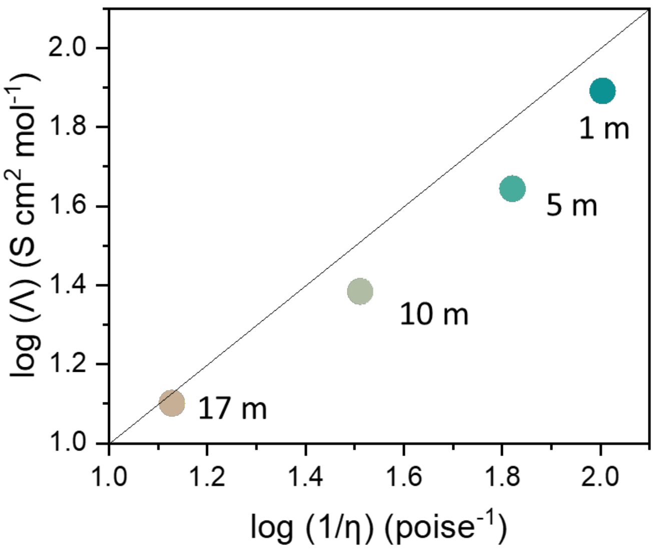

Figure S6. Walden plot for $NaClO_4$ electrolytes.

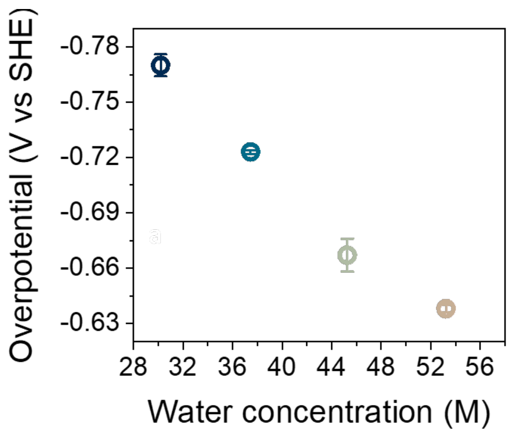

Figure S7. Overpotential to reach 1mA/cm^2 *vs* water concentration in mol-per-liter.

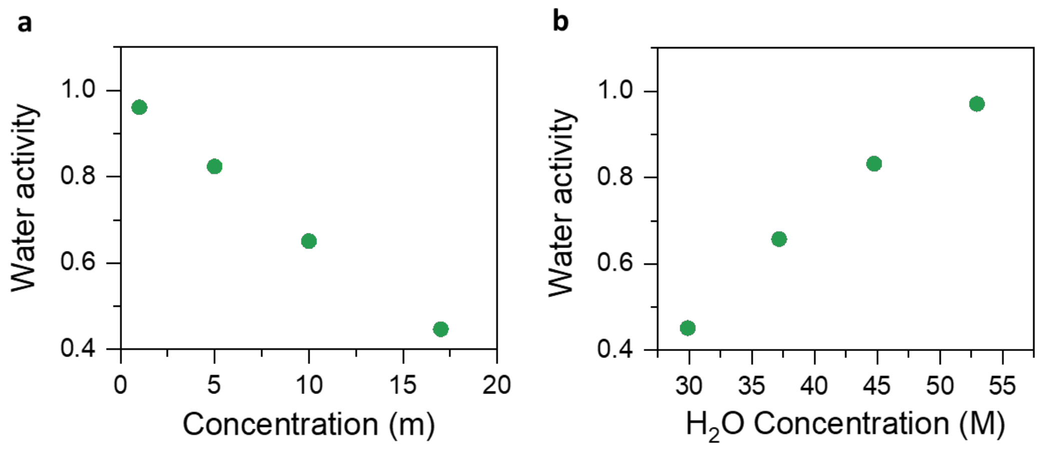

Figure S8. Measured water activity from the equilibrium with gas phase for the electrolytes at different concentrations. a) activity as a fraction of 1 vs electrolyte concentration. b) activity vs real water concentration molarity.

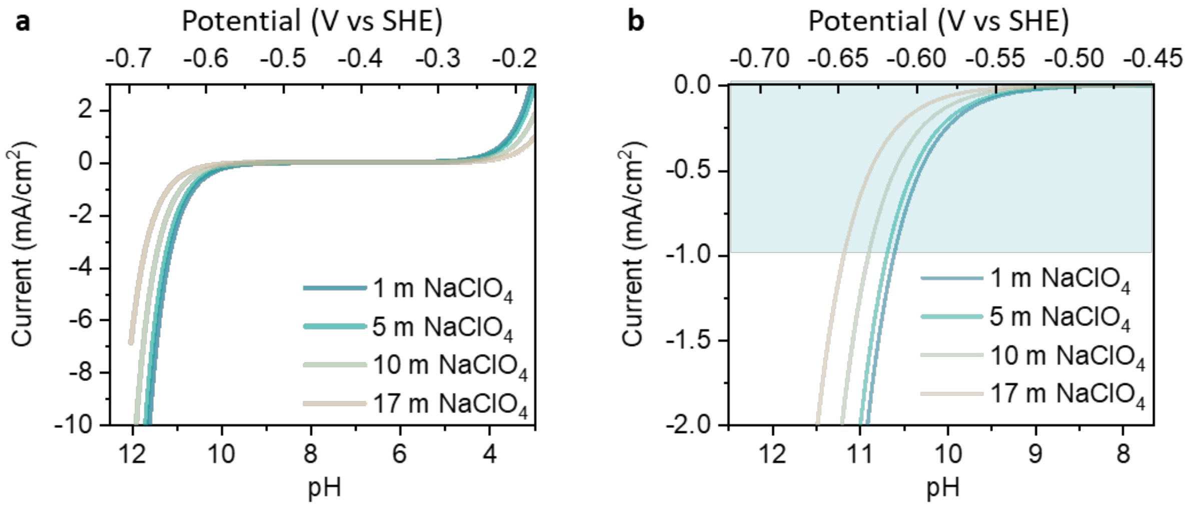

Figure S9. Local pH shift as function of current for HER and HOR in different concentrations. (a) The local pH and the corresponding onset potential for RHE vs SHE for the different electrolyte concentrations. (b) Zoom-in region of the LSV (blue region depicts currents <=1 mA/cm^2).

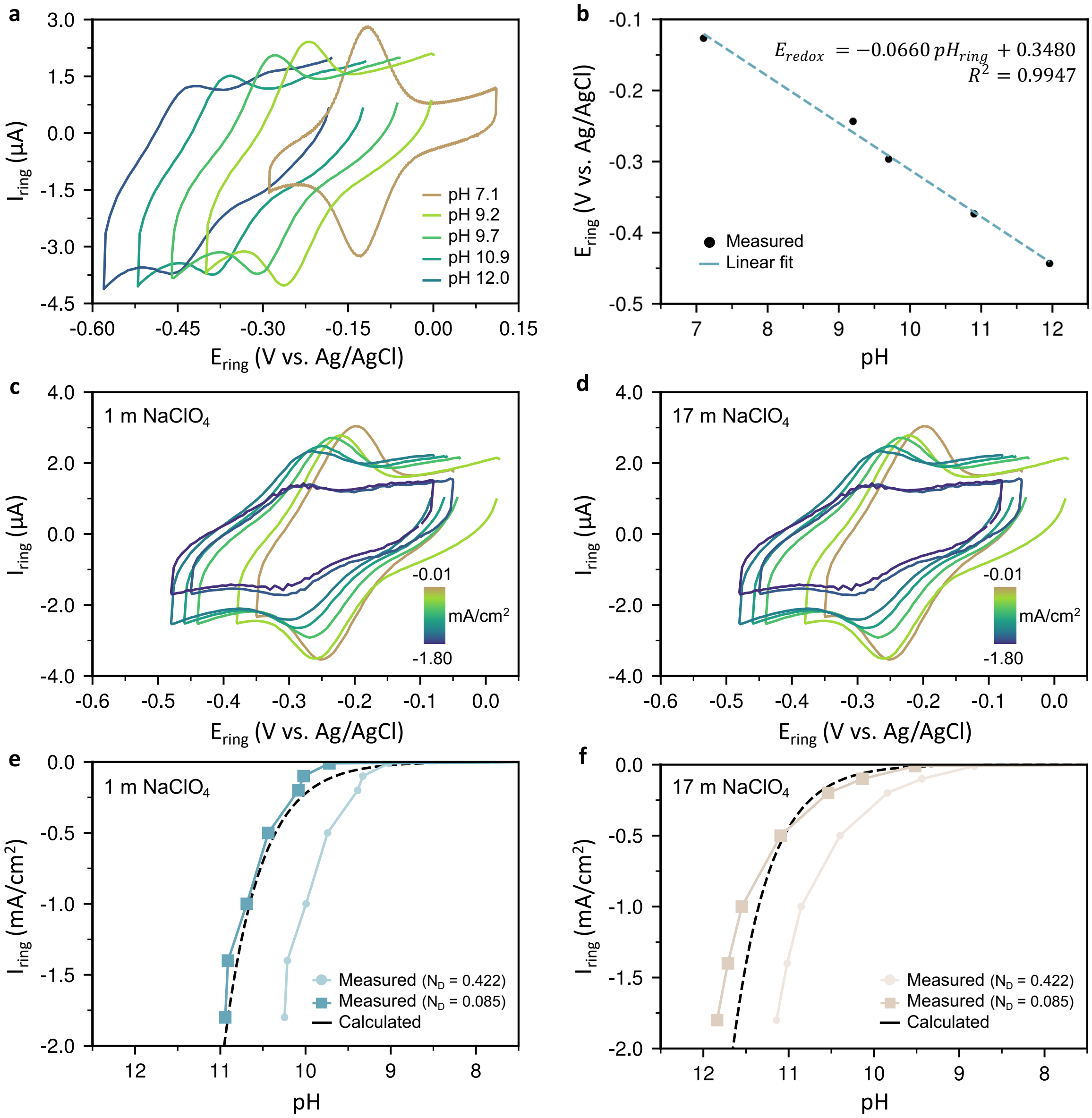

Figure S10. In-situ HER local pH measurement collected in a rotating Ring-disk electrode (RRDE) configuration at 1600 rpm through step chrono amperometry. (a) Ring CVs in different pH calibration solutions. (b) Linear fit of redox potentials from calibration. (c)-(d) Ring CVs at different disk current densities. (e)-(f) Calculated and measured local pH as a function of geometric current density for 1 and 17 m $NaClO_4$ with N_D of 42.2% or corrected N_D of 8.5%.

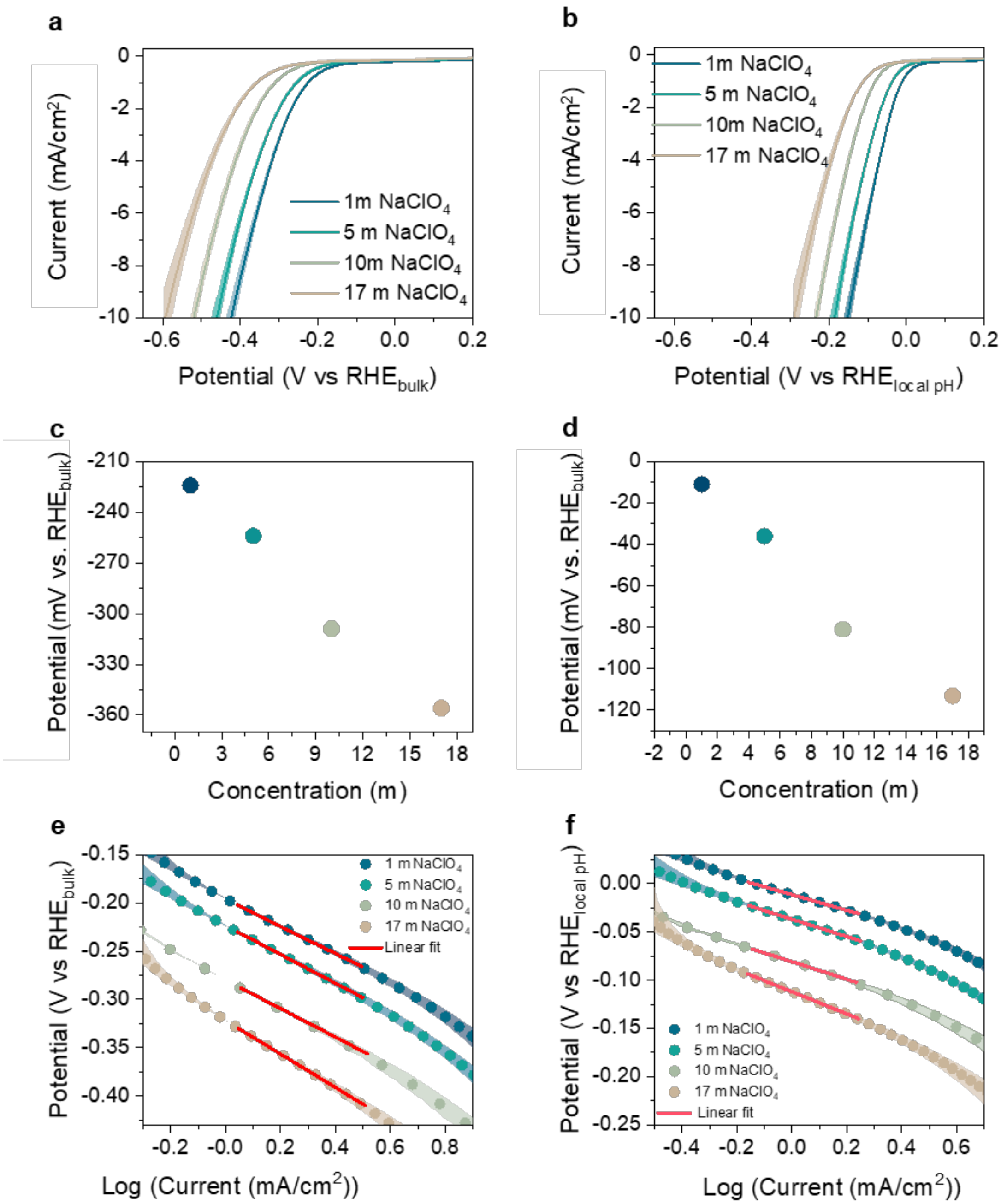

Figure S11. HER LSV corrected for local pH at the surface of the electrode in V vs RHE. (a) LSV with no correction vs RHE considering bulk pH, (b) LSV vs RHE considering local pH, (c) Overpotential to reach 1 mA/cm^2 considering bulk pH, (d) Overpotential to reach 1 mA/cm^2 considering local pH, (e) Tafel slopes before correction considering bulk pH. (f) Tafel slopes considering local pH at the electrode.

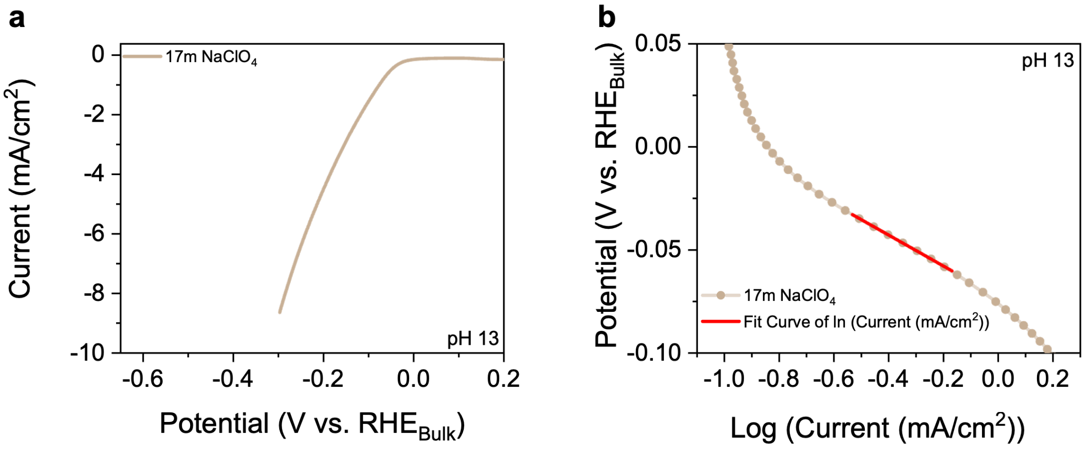

Figure S12. HER measurement in 17 m $NaClO_4$ + 0.1 M NaOH (0.18 m NaOH) solution adjusted to pH 13: (a) Linear sweep voltammetry, (b) Tafel slope.

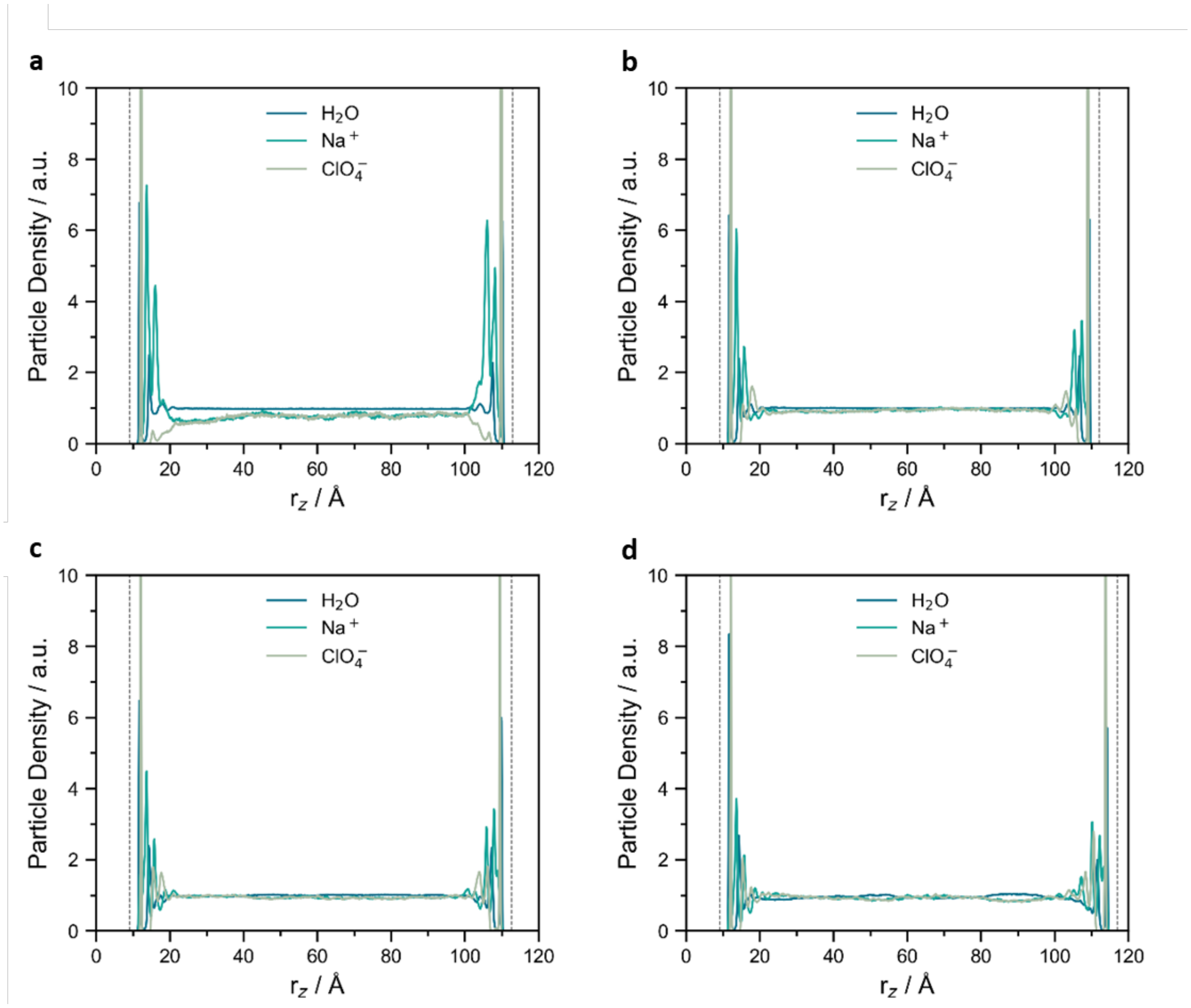

Figure S13. Average particle density along the z axis relative to the total density at salt concentrations of 1 m (a), 5 m (b), 10 m (c), and 17 m (d) at 1 V potential. Dashed lines indicate the electrode surfaces.

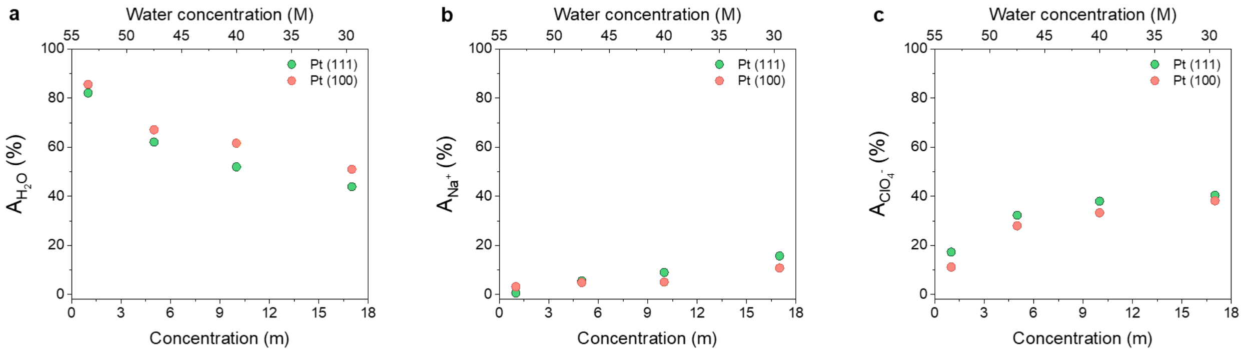

Figure S14. MD extracted surface speciation at Pt (111) and Pt (100) electrodes at 1V polarization. (a)-(c) population changes as function of concentration.

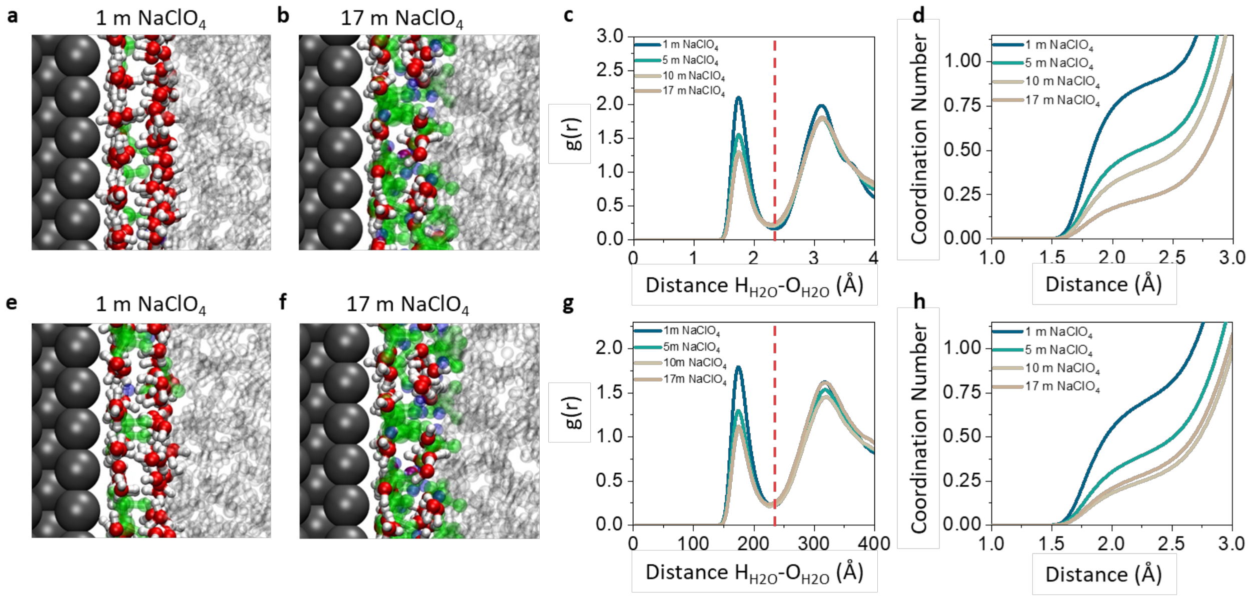

Figure S15. Interface Bilayer structure at a Pt (100) and Pt (111) electrode. (a)-(b) Pt (100) Bilayer visualization extracted from MD at different concentrations. (c) G(r) H_{H2O} – O_{H2O} for the first water monolayer at Pt (100) electrode. (d) Coordination number for H_{H2O} – O_{H2O} pair at the Pt (100) electrode. (e)-(f) Pt (111) Bilayer visualization extracted from MD at different concentrations. (g) G(r) H_{H2O} – O_{H2O} for the first water monolayer at Pt (111) electrode. (h) Coordination number for H_{H2O} – O_{H2O} pair at Pt (111) electrode.

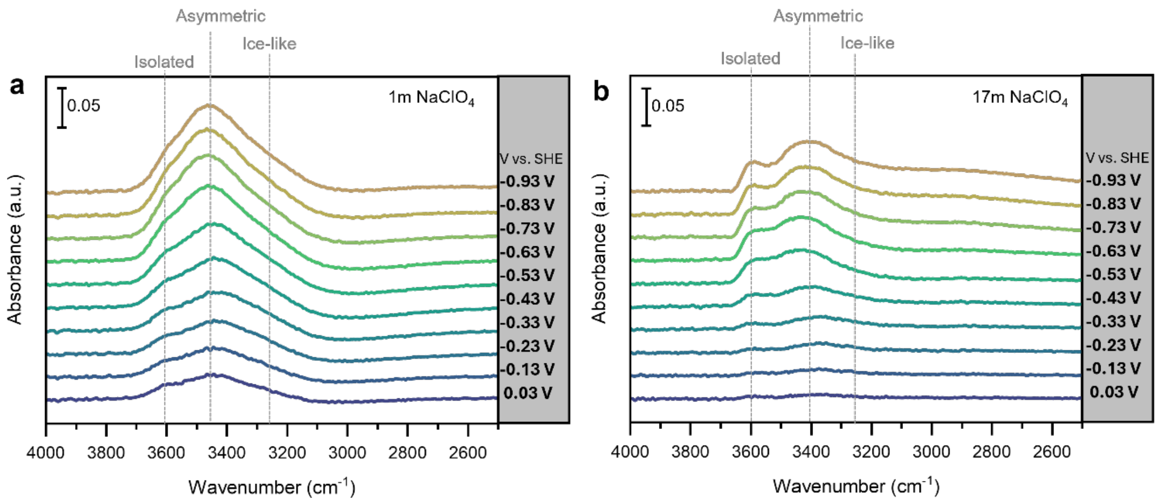

Figure S16. SEIRAS spectra of the v(O-H) region during linear sweep voltammetry (10 mV/s) in 1m $NaClO_4$ (a) and 17m $NaClO_4$ (b). A spectrum was collected every 10s and corresponds to a voltage range of 100 mV. The lower potential limit of the voltage range was denoted next to the respective spectrum. The spectra for 1m and 17 m $NaClO_4$ were both collected on the same Pt thin film electrode with a background acquisition at 0.23V vs SHE.

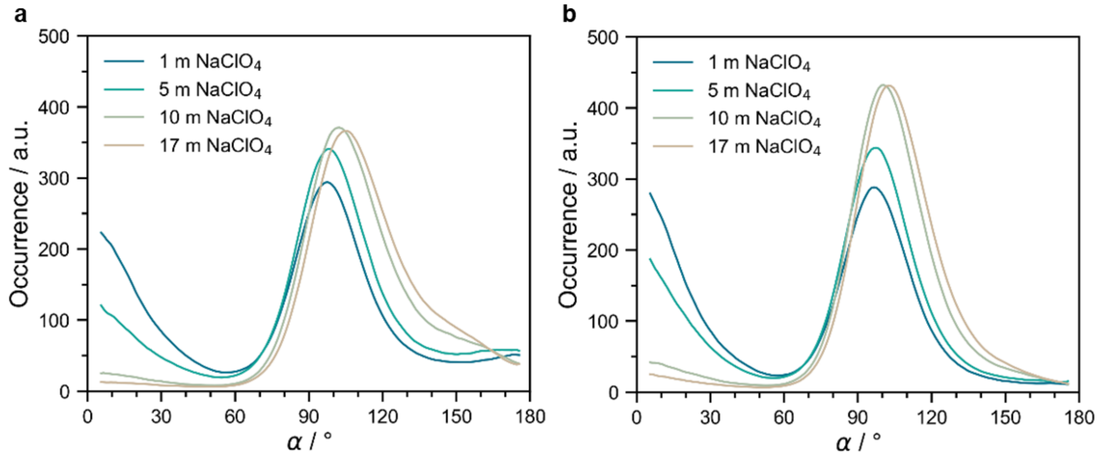

Figure S17. Angular distribution function (ADF) of the O-H bond of water molecules in the cathode's first adlayer against the z axis, where α = 0° and α = 180° indicate O-H bonds pointing away from and towards the electrode surface, respectively. (a) ADF measured at the cathode surface under a potential of 1 V. (b) ADF measured at the cathode surface under a potential of 0 V.

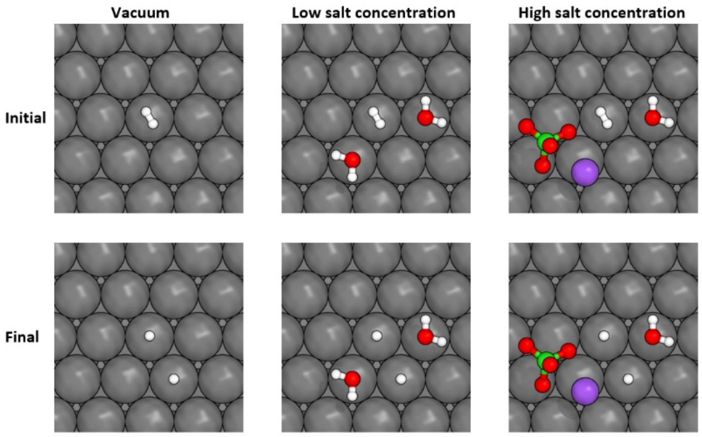

Figure S18. Initial and final states for the NEB calculations of the Volmer step at different environments.

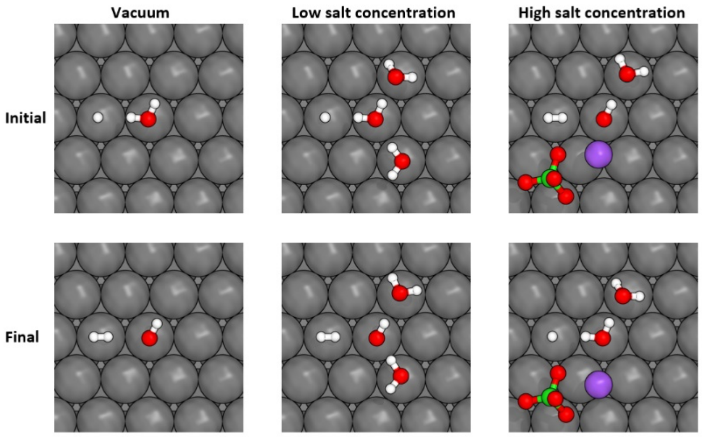

Figure S19. Initial and final states for the NEB calculations of the Heyrovski step at different environments.

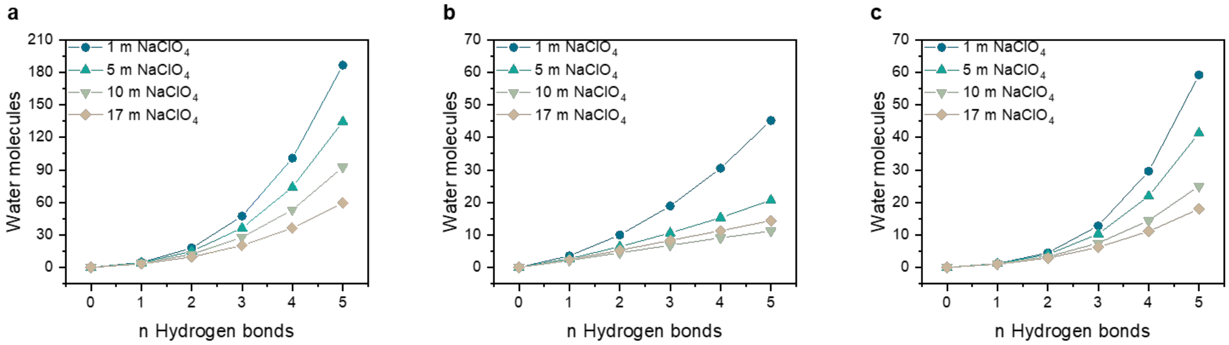

Figure S20. Average number of water molecules connected through n hydrogen bonds for (a) water molecules in the bulk, (b) water molecules adsorbed to the cathode surface counting only horizontal hydrogen bonds (parallel to the surface), (c) water molecules adsorbed to the cathode surface excluding horizontal hydrogen bonds (normal to the surface).

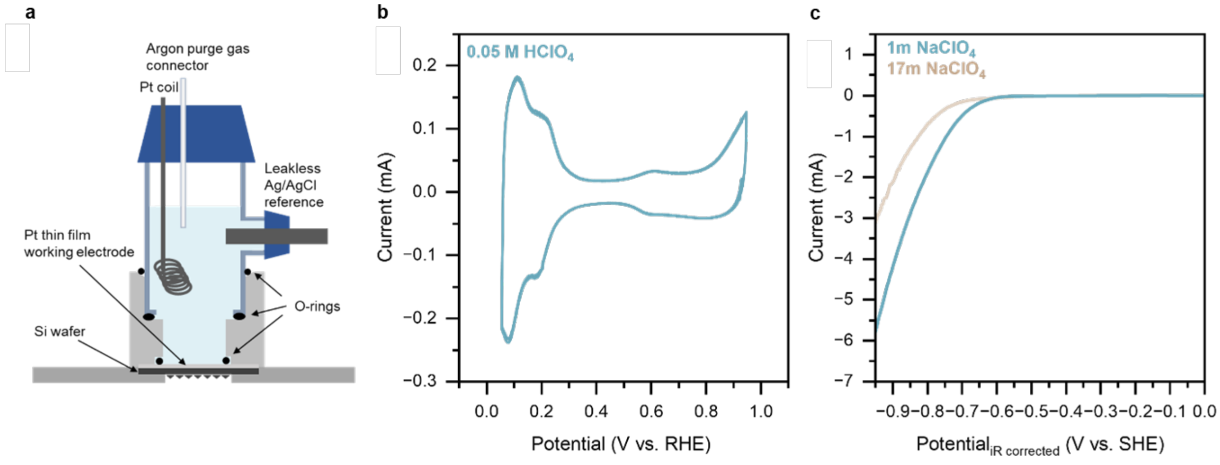

Figure S21. Electrochemical set-up and experimental data for in-situ SEIRAS measurements (a) cyclic voltammetry of the electrodeposited Pt thin film (100 mV/s). The absence of a gold reduction peak indicates the pinhole-free coverage of the gold underlayer. (b) Current response during linear sweep voltammetry (10 mV/s) in the respective sodium perchlorate electrolyte during SEIRAS measurements. (c) Drawing of the used FTIR cell for SEIRAS measurements.

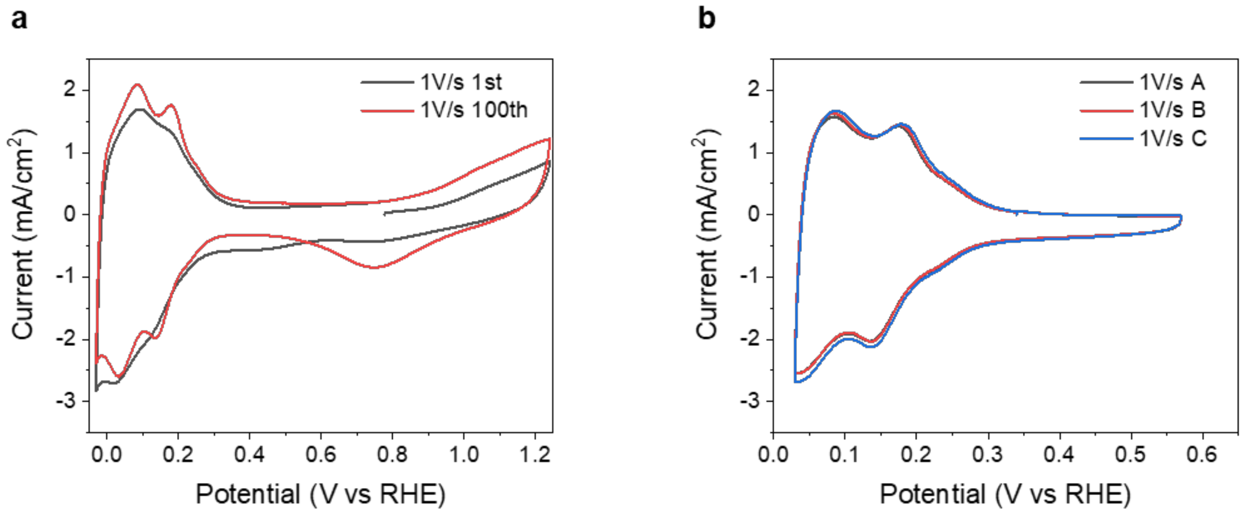

Figure S22. Electrochemical cleaning in 50 mM $HClO_4$. (a) shows the CVs from 0 -1.2 V vs RHE for polycrystalline Pt for the first and 100th cycle collected at 1000 mV/s. (b) shows the H-UPD CV from the potential 60 mV to 600 mV vs RHE for electrochemical surface area determination collected at 1000 mV/s.

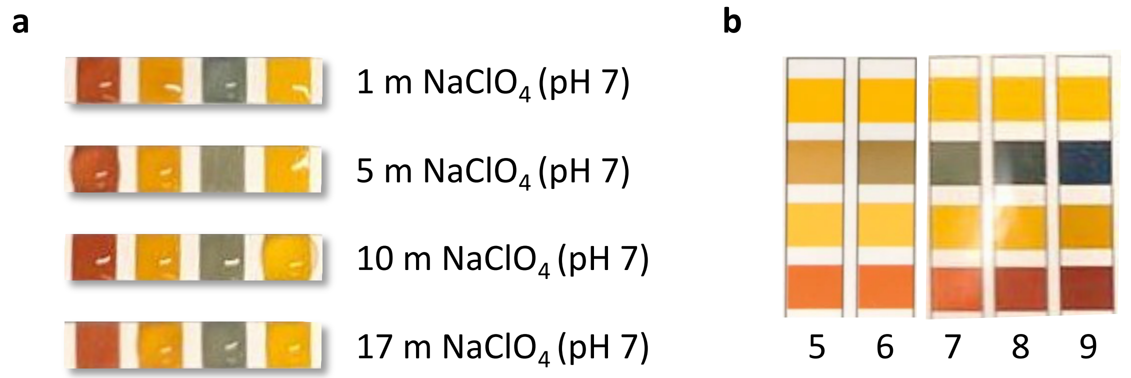

Figure S23. Measurement of bulk pH using pH indicator strips. (a) shows the resulting pH indicator strips after being submerged in the $NaClO_4$ solutions. (b) provides the pH indicator key as provided on the pH strip package.

SUPPLEMENTARY TABLES

Table S1. Bulk pH measurements with pH meter and pH indicator strips

**Concentration molal**	**pH (measured with pH meter)**	**pH (measured with pH strips)**
1	6.85 ± 0.20	7
5	6.71 ± 0.21	7
10	6.74 ± 0.25	7
17	7.12 ± 0.21	7

Table S2. Tafel slopes fitting results when considering bulk and local pH from the numerical model

	Bulk pH				Local pH				
**Concentration**	**slope**	**error**	**Intercept**	**error**	**slope**	**error**	**Intercept**	**error**	**Local pH**
**molal**	**mV/dec**	**mV/dec**	**mA/cm^2**	**mA/cm^2**	**mV/dec**	**mV/dec**	**mA/cm^2**	**mA/cm^2**	
1	-139.97	0.41	0.023	-7.9E-05	-81.46	0.43	0.724	0.004	10.63
5	-146.87	0.45	0.018	-6.2E-05	-91.13	0.43	0.394	0.002	10.77
10	-151.02	0.96	0.008	-5.8E-05	-91.11	1.72	0.129	0.002	11.02
17	-173.22	0.48	0.008	-2.5E-05	-115.49	0.57	0.107	0.001	11.33
17 (pH 13)	-75.09	0.20	0.107	0.000	-75.09	0.20	0.107	0.000	13(≈bulk)

Table S3. Composition and final box dimensions of the simulated systems. d_{el} is the distance between electrode surfaces.

**Bulk**	**1 m**	**5 m**	**10 m**	**17 m**
**H_2O**	6450	5800	5200	4500
**Na**	116	522	936	1378
**ClO4**	116	522	936	1378
**a / Å**	58.6963	59.5437	60.7011	61.8789
**ρ / g/cm³**	1.0708	1.3246	1.5464	1.7506
**Pt(100)**				
**H_2O**	5500	4700	4000	3400
**Na**	100	424	720	1041
**ClO_4**	100	424	720	1041
**Pt**	2560	2560	2560	2560
**a / Å**	44.3497	44.3497	44.3497	44.3497
**b / Å**	44.3497	44.3497	44.3497	44.3497
**c / Å**	105.9371	105.2079	105.6721	109.3752
**d_{el} / Å**	90.2571	89.5279	89.9921	93.6952
**Pt(111)**				
**H_2O**	5500	4700	4000	3400
**Na**	100	424	720	1041
**ClO_4**	100	424	720	1041
**Pt**	2560	2560	2560	2560
**a / Å**	44.3497	44.3497	44.3497	44.3497
**b / Å**	38.4080	38.4080	38.4080	38.4080
**c / Å**	121.9389	121.0968	121.6328	125.9089
**d_{el} / Å**	103.8331	102.9912	103.5272	107.8031

SUPPLEMENTARY REFERENCES

1. Zhao, Y.; Hu, X.; Stucky, G. D.; Boettcher, S. W., Thermodynamic, Kinetic, and Transport Contributions to Hydrogen Evolution Activity and Electrolyte-Stability Windows for Water-in-Salt Electrolytes. *Journal of the American Chemical Society* **2024,** *146* (5), 3438-3448.
2. Čolić, V.; Tymoczko, J.; Maljusch, A.; Ganassin, A.; Schuhmann, W.; Bandarenka, A. S., Experimental Aspects in Benchmarking of the Electrocatalytic Activity. *ChemElectroChem* **2015,** *2* (1), 143-149.
3. Chen, Q.-S.; Solla-Gullón, J.; Sun, S.-G.; Feliu, J. M., The potential of zero total charge of Pt nanoparticles and polycrystalline electrodes with different surface structure: The role of anion adsorption in fundamental electrocatalysis. *Electrochimica Acta* **2010,** *55* (27), 7982-7994.
4. Auinger, M.; Katsounaros, I.; Meier, J. C.; Klemm, S. O.; Biedermann, P. U.; Topalov, A. A.; Rohwerder, M.; Mayrhofer, K. J. J., Near-surface ion distribution and buffer effects during electrochemical reactions. *Physical Chemistry Chemical Physics* **2011,** *13* (36), 16384-16394.
5. Baer, A.; Wawra, S. E.; Bielmeier, K.; Uttinger, M. J.; Smith, D. M.; Peukert, W.; Walter, J.; Smith, A.-S., The Stokes–Einstein–Sutherland Equation at the Nanoscale Revisited. *Small* **2024,** *20* (6), 2304670.
6. Liu, X.; Monteiro, M. C. O.; Koper, M. T. M., Interfacial pH measurements during CO2 reduction on gold using a rotating ring-disk electrode. *Physical Chemistry Chemical Physics* **2023,** *25* (4), 2897-2906.
7. Albery, W. J.; Bruckenstein, S., Ring-disc electrodes. Part 2.—Theoretical and experimental collection effciencies. *Transactions of the Faraday Society* **1966,** *62* (0), 1920-1931.
8. Thompson, A. P.; Aktulga, H. M.; Berger, R.; Bolintineanu, D. S.; Brown, W. M.; Crozier, P. S.; in 't Veld, P. J.; Kohlmeyer, A.; Moore, S. G.; Nguyen, T. D.; Shan, R.; Stevens, M. J.; Tranchida, J.; Trott, C.; Plimpton, S. J., LAMMPS - a flexible simulation tool for particle-based materials modeling at the atomic, meso, and continuum scales. *Computer Physics Communications* **2022,** *271*, 108171.
9. Jensen, K. P.; Jorgensen, W. L., Halide, Ammonium, and Alkali Metal Ion Parameters for Modeling Aqueous Solutions. *Journal of Chemical Theory and Computation* **2006,** *2* (6), 1499-1509.
10. Doherty, B.; Zhong, X.; Gathiaka, S.; Li, B.; Acevedo, O., Revisiting OPLS Force Field Parameters for Ionic Liquid Simulations. *Journal of Chemical Theory and Computation* **2017,** *13* (12), 6131-6145.
11. Berendsen, H. J. C.; Grigera, J. R.; Straatsma, T. P., The missing term in effective pair potentials. *The Journal of Physical Chemistry* **1987,** *91* (24), 6269-6271.
12. Lennard-Jones, J. E., Cohesion. *Proceedings of the Physical Society* **1931,** *43* (5), 461.
13. Martínez, L.; Andrade, R.; Birgin, E. G.; Martínez, J. M., PACKMOL: A package for building initial configurations for molecular dynamics simulations. *Journal of Computational Chemistry* **2009,** *30* (13), 2157-2164.
14. Braga, C.; Travis, K. P., A configurational temperature Nosé-Hoover thermostat. *The Journal of Chemical Physics* **2005,** *123* (13), 134101.
15. Hoover, W. G.; Posch, H. A.; Holian, B. L.; Gillan, M. J.; Mareschal, M.; Massobrio, C., Dissipative Irreversibility from Nosé's Reversible Mechanics. *Molecular Simulation* **1987,** *1* (1-2), 79-86.
16. Ahrens-Iwers, L. J. V.; Janssen, M.; Tee, S. R.; Meißner, R. H., ELECTRODE: An electrochemistry package for atomistic simulations. *The Journal of Chemical Physics* **2022,** *157* (8), 084801.
17. Heinz, H.; Vaia, R. A.; Farmer, B. L.; Naik, R. R., Accurate Simulation of Surfaces and Interfaces of Face-Centered Cubic Metals Using 12−6 and 9−6 Lennard-Jones Potentials. *The Journal of Physical Chemistry C* **2008,** *112* (44), 17281-17290.
18. Hollóczki, O.; Macchiagodena, M.; Weber, H.; Thomas, M.; Brehm, M.; Stark, A.; Russina, O.; Triolo, A.; Kirchner, B., Triphilic Ionic-Liquid Mixtures: Fluorinated and Non-fluorinated Aprotic Ionic-Liquid Mixtures. *ChemPhysChem* **2015,** *16* (15), 3325-3333.

19. Brehm, M.; Kirchner, B., TRAVIS - A Free Analyzer and Visualizer for Monte Carlo and Molecular Dynamics Trajectories. *Journal of Chemical Information and Modeling* **2011,** *51* (8), 2007-2023.
20. Brehm, M.; Thomas, M.; Gehrke, S.; Kirchner, B., TRAVIS—A free analyzer for trajectories from molecular simulation. *The Journal of Chemical Physics* **2020,** *152* (16), 164105.
21. Bondi, A., van der Waals Volumes and Radii. *The Journal of physical chemistry* **1964,** *68* (3), 441-451.
22. Kühne, T. D.; Iannuzzi, M.; Del Ben, M.; Rybkin, V. V.; Seewald, P.; Stein, F.; Laino, T.; Khaliullin, R. Z.; Schütt, O.; Schiffmann, F.; Golze, D.; Wilhelm, J.; Chulkov, S.; Bani-Hashemian, M. H.; Weber, V.; Borštnik, U.; Taillefumier, M.; Jakobovits, A. S.; Lazzaro, A.; Pabst, H.; Müller, T.; Schade, R.; Guidon, M.; Andermatt, S.; Holmberg, N.; Schenter, G. K.; Hehn, A.; Bussy, A.; Belleflamme, F.; Tabacchi, G.; Glöß, A.; Lass, M.; Bethune, I.; Mundy, C. J.; Plessl, C.; Watkins, M.; VandeVondele, J.; Krack, M.; Hutter, J., CP2K: An electronic structure and molecular dynamics software package - Quickstep: Efficient and accurate electronic structure calculations. *The Journal of Chemical Physics* **2020,** *152* (19), 194103.
23. Sha, Y.; Yu, T. H.; Merinov, B. V.; Goddard, W. A., III, DFT Prediction of Oxygen Reduction Reaction on Palladium–Copper Alloy Surfaces. *ACS Catalysis* **2014,** *4* (4), 1189-1197.
24. Mills, G.; Jónsson, H., Quantum and thermal effects in ${\mathrm{H}}_{2}$ dissociative adsorption: Evaluation of free energy barriers in multidimensional quantum systems. *Physical Review Letters* **1994,** *72* (7), 1124-1127.
25. Mills, G.; Jónsson, H.; Schenter, G. K., Reversible work transition state theory: application to dissociative adsorption of hydrogen. *Surface Science* **1995,** *324* (2), 305-337.
26. Goedecker, S.; Teter, M.; Hutter, J., Separable dual-space Gaussian pseudopotentials. *Physical Review B* **1996,** *54* (3), 1703-1710.
27. Hartwigsen, C.; Goedecker, S.; Hutter, J., Relativistic separable dual-space Gaussian pseudopotentials from H to Rn. *Physical Review B* **1998,** *58* (7), 3641-3662.
28. Krack, M., Pseudopotentials for H to Kr optimized for gradient-corrected exchange-correlation functionals. *Theoretical Chemistry Accounts* **2005,** *114* (1), 145-152.
29. Grimme, S.; Antony, J.; Ehrlich, S.; Krieg, H., A consistent and accurate ab initio parametrization of density functional dispersion correction (DFT-d) acta morphologica hungaricafor the 94 elements H-Pu. *J. Chem. Phys* **2010,** *132*, 15.
30. Grimme, S.; Ehrlich, S.; Goerigk, L., Effect of the damping function in dispersion corrected density functional theory. *Journal of Computational Chemistry* **2011,** *32* (7), 1456-1465.
31. Zhang, J.; Dolg, M., ABCluster: the artificial bee colony algorithm for cluster global optimization. *Physical Chemistry Chemical Physics* **2015,** *17* (37), 24173-24181.
